# Functionalized Amyloid‐Like Protein Nanofilm‐Mediated Synergistic Disulfidptosis and Photodynamic Therapy for Preventing Postoperative Recurrence of Colorectal Cancer

**DOI:** 10.1002/smsc.202400323

**Published:** 2024-09-23

**Authors:** Man Zhang, Ke Li, Junhao Kou, Guozhi Lu, Ling Qiu, Chunzhao Yang, Yongchun Liu, Qi Xue, Peng Yang

**Affiliations:** ^1^ Department of General Surgery Southern Medical University Hospital of Integrated Traditional Chinese and Western Medicine Southern Medical University Guangdong 510315 China; ^2^ Xi'an Key Laboratory for Prevention and Treatment of Common Aging Diseases Translational and Research Centre for Prevention and Therapy of Chronic Disease Institute of Basic and Translational Medicine Xi'an Medical University Xi'an 710021 China; ^3^ College of Pharmacy Xi'an Medical University Xi'an 710021 China; ^4^ College of Clinical Medicine Xi'an Medical University Xi'an 710021 China; ^5^ Key Laboratory of Applied Surface and Colloid Chemistry Ministry of Education School of Chemistry and Chemical Engineering Shaanxi Normal University Xi'an 710119 China

**Keywords:** 2D protein film, colorectal cancer recurrence, disulfidptosis, synergism, wet interface adhesion

## Abstract

Colorectal cancer (CRC) has a postoperative recurrence rate of up to 50%. Local treatment at the surgical site offers a new strategy to prevent recurrence, minimizing systemic side effects. However, these treatments often face issues like poor stability, insufficient targeting, and significant local side effects. Research shows glucose starvation can induce disulfidptosis, a novel cell death pathway, in CRC cells, suitable for precise intervention in residual tumor cells postsurgery. This study develops a biocompatible 2D therapeutic platform using amyloid‐like protein nanofilm technology for precise local synergistic intervention in disulfidptosis and photodynamic therapy (PDT) after CRC surgery. The nanofilm boasts excellent drug‐loading capacity, enzyme activity retention, ultrathin dimensions, wet adhesion, and biocompatibility. A model simulating incomplete tumor resection post‐CRC surgery shows that the GCI@nanofilm effectively inhibits recurrence through an enzyme‐catalyzed cascade reaction inducing disulfidptosis and synergizing with PDT. In summary, GCI@nanofilm overcomes multiple challenges in local postsurgical intervention, providing a foundation for precise and efficient treatment of residual tumors.

## Graphical abstract

Colorectal cancer has a high postoperative recurrence rate. This study introduces a biocompatible 2D therapeutic platform using amyloid‐like protein nanofilm technology to enhance local treatment efficacy. The GCI@nanofilm induces disulfidptosis and synergizes with PDT, effectively inhibiting tumor recurrence in models of incomplete resection. This approach addresses challenges in local interventions for residual tumors.

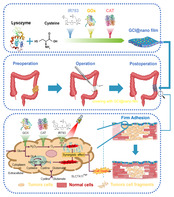

## 1 Introduction

Colorectal cancer (CRC) is the third‐most prevalent cancer globally and has the second‐highest mortality rate.^[^ ^1^ ^]^ Due to its insidious onset, a majority of patients are diagnosed at an advanced stage where complete tumor resection becomes challenging, resulting in a high recurrence rate of up to 50%.^[^ ^2^ ^]^ Postoperative adjuvant chemotherapy serves as the primary approach to prevent recurrence; however, it not only involves significant side effects but also can only undergo the procedure 3–4 weeks after surgery, depending on their physical condition. This timing often misses the optimal window to eliminate residual tumor cells and prevent recurrence.^[^ ^3^ ^]^ Thus, the exploration of novel strategies to precisely prevent recurrence remains a key focus in CRC clinical research.^[^ ^4^ ^]^ Tumor exhibits a unique metabolic profile distinct from normal tissues, rendering them susceptible to unconventional cell death pathways.^[^ ^5^ ^]^ In recent years, many scholars have regulated cell death by modulating tumor metabolism, such as ferroptosis and cuproptosis.^[^ ^6^ ^]^ Particularly, disulfidptosis, a newly defined form of cell death by Gan et al. in 2023, involves the high expression of SLC7A11 in cells under glucose‐starvation conditions, depletes intracellular NADPH, and leads to the abnormal accumulation of disulfides like cysteine, ultimately triggering rapid cell death mediated by disulfide stress.^[^ ^7^ ^]^ Notably, the gene expression of SLC7A11 in CRC tissues significantly exceeds that in normal tissues, establishing a conducive tumor microenvironment for inhibiting the cancer by activating disulfidptosis. Moreover, this method of inducing cell death through abnormal metabolism offers advantages such as high specificity and minimal side effects compared to drug therapies, making it highly suitable for direct prevention of CRC recurrence postoperatively.

**Scheme 1:**
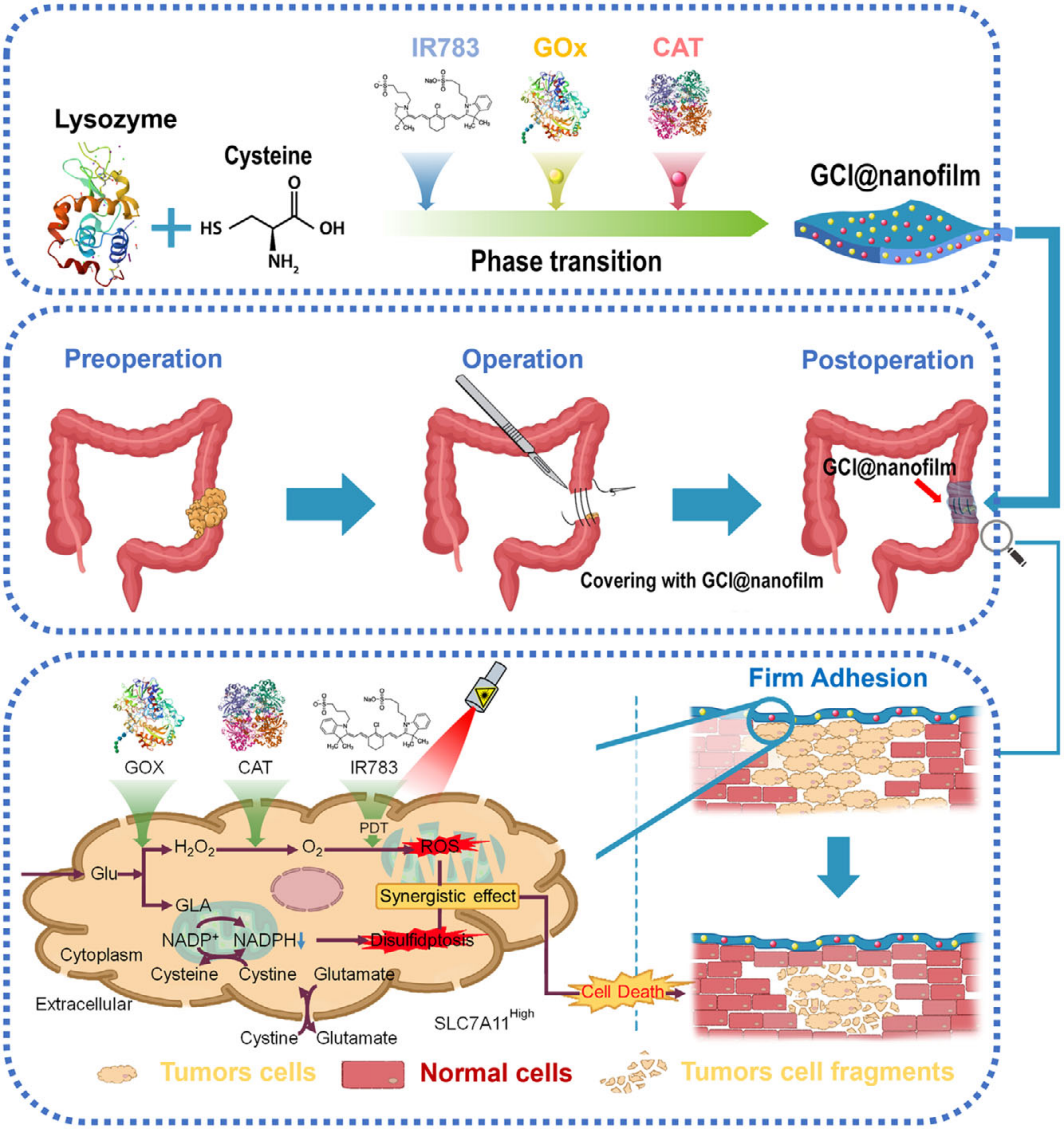
Schematic illustration of the synthesis of GCI@nanofilm, the establishment of an orthotopic CRC incomplete resection model, and the synergistic treatment process of GCI@nanofilm‐induced disulfidptosis and PDT for CRC recurrence.

Direct local drug administration to the tumor site immediately after resection during surgery can enhance treatment efficiency and reduce systemic toxicity by directly applying therapeutic molecules to residual tumor areas. This approach is particularly beneficial for suppressing CRC recurrence.^[^ ^8^ ^]^ The key to achieving local administration lies in how to ensure that the therapeutic molecules continuously and efficiently act on the lesion site.^[^ ^9^ ^]^ In recent years, various emerging drug delivery systems, including injectable hydrogels,^[^ ^10^ ^]^ implantable stents, polymer sponges, and electrospinning, are employed in postoperative drug delivery for malignant tumor.^[^ ^11^ ^]^ However, these systems often encounter challenges during treatment. Issues such as poor adhesion to wet and motile tissue interfaces leading to detachment, inadequate contact of the relatively thick and rigid substrate with the tissue, and mismatched degradation or drug release rates can result in suboptimal or ineffective local drug administration.^[^ ^12^ ^]^ Furthermore, for these materials, multifunctional integration may not be challenging; however, the bottleneck lies in achieving efficient synergistic effects after integration.^[^ ^13^ ^]^ Efficient synergy of multiple therapeutic methods is crucial for effectively increasing the clearance rate of cancer cells and reducing the number of residual cancer cells, thereby lowering the risk of recurrence.^[^ ^14^ ^]^ Exploring suitable drug delivery materials is crucial in addressing these challenges. Previous studies conducted by our research group have demonstrated that breaking disulfide bonds in proteins yield stable 2D nanofilms assembled at interfaces.^[^ ^15^ ^]^ These protein nanofilms exhibit excellent strong wet surface adhesion to various tissues and can load various bioactive molecules, serving as a platform for drug release and multifunctional synergy, aligning well with the requirements of local in situ treatment post‐CRC surgery (**Scheme** **1**).^[^ ^16^ ^]^

Therefore, this study utilizes a 2D protein film as the functional platform to achieve disulfidptosis‐induced synergistic photodynamic therapy (PDT) for preventing recurrence after CRC surgery. Glucose oxidase (GOx) and catalase (CAT) enzymes, along with the photosensitizer IR783, are combined with lysozyme to rapidly fabricate a GOx/CAT/IR783‐functionalized protein nanofilm (GCI@nanofilm) using a mild one‐pot method.^[^ ^17^ ^]^ GOx catalyzes the oxidation of glucose to produce gluconic acid and hydrogen peroxide disrupting the glucose supply to tumor cells, down‐regulating NADPH, and initiating disulfidptosis. Subsequently, its catalytic product, hydrogen peroxide, is catalyzed by the loaded enzyme CAT to generate oxygen, thereby alleviating the tumor's hypoxic microenvironment. This provides oxygen substrates for the IR783 PDT, further increasing the formation of reactive oxygen species (ROS) and achieving synergistic treatment.^[^ ^18^ ^]^ The study simulates the surgical resection scenario to establish an orthotopic CRC incomplete resection model, effectively validating the therapeutic effect of GCI@nanofilm. This study lays an experimental foundation for exploring new methods for the treatment of advanced CRC.^[^ ^19^ ^]^

## 2 Results

### 2.1 Characterization of the GCI@nanofilm

**Figure 1:**
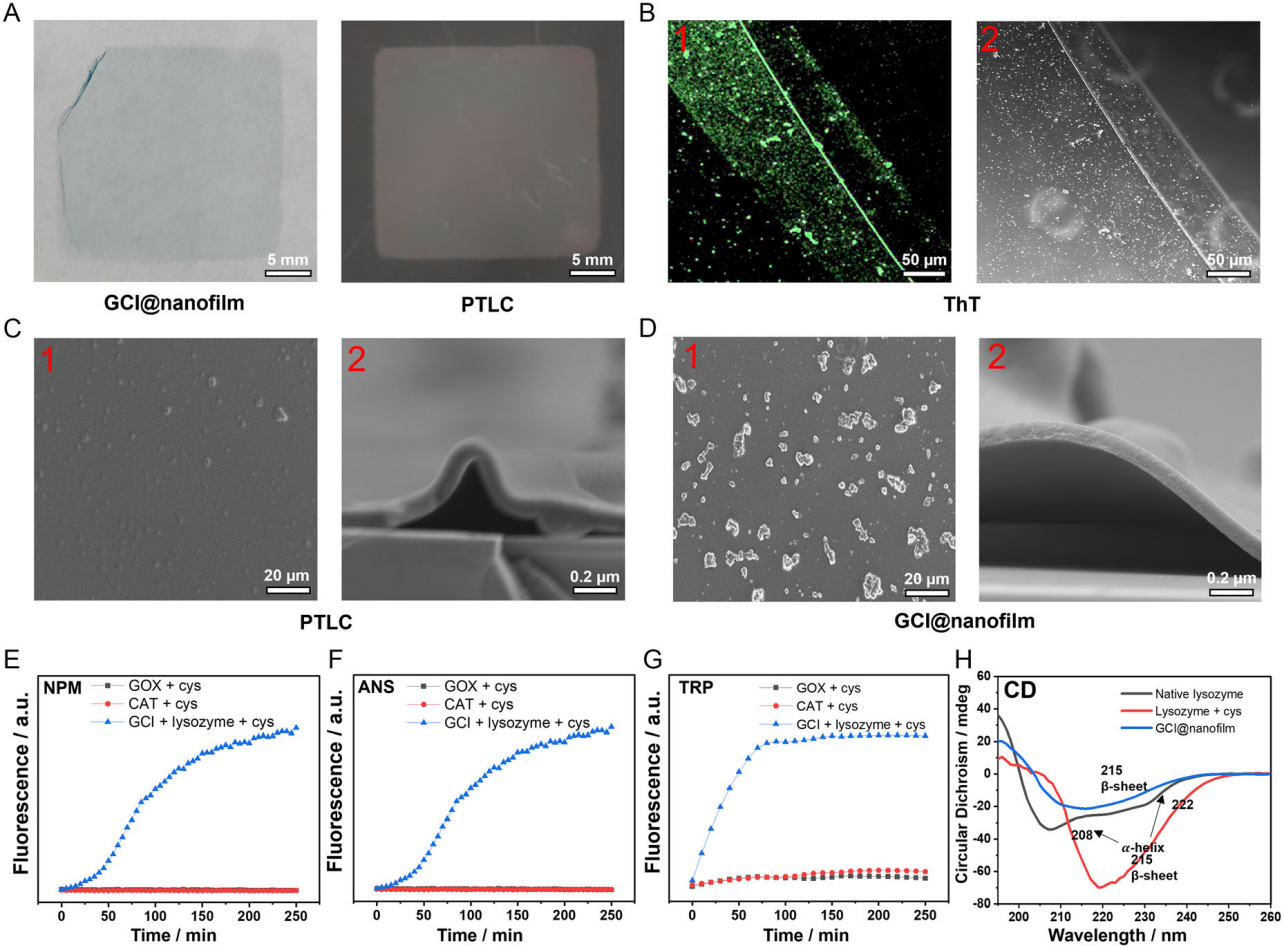
Characterization of the GCI@nanofilm. A) Optical images of GCI@nanofilm and PTLC. B) Confocal surface images of ThT‐stained GCI@nanofilm, scale = 50 μm, B1 shows a confocal microscopy image, and B2 shows an optical image, scale = 50 μm. C,D) SEM planar and cross‐sectional views of GCI@nanofilm and PTLC, C1, D1 scale = 20 μm, C2, D2, scale = 0.2 μm. E) NPM fluorescence intensity over time for GOx and cysteine, CAT and cysteine, GOx, CAT, IR783, lysozyme, and cysteine. F) ANS fluorescence intensity over time for GOx and cysteine, CAT and cysteine, GOx, CAT, IR783, lysozyme, and cysteine. G) Trp fluorescence intensity over time for GOx and cysteine, CAT and cysteine, GOx, CAT, IR783, lysozyme, and cysteine. H) CD spectra of native lysozyme, native lysozyme with cysteine (nanofilm), and GCI@nanofilm (nanofilm).

A series of characterization methods are employed to confirm that the GCI@nanofilm, encapsulating GOx, CAT, and IR783, successfully forms a 2Dl protein film with excellent adhesion, mechanical properties, biocompatibility, and stability. As reported by our previous studies, the selective disruption of disulfide bonds (Cys6‐Cys127) in natural lysozyme by cysteine induces partial unfolding of the protein, spontaneously transforming from a high‐energy α‐helix state to a low‐energy β‐sheet state.^[^ ^20^ ^]^ This process leads to the self‐assembly of large‐sized protein nanofilms or protein coatings at interfaces.^[^ ^21^ ^]^ Scanning electron microscope (SEM) images reveal that the surface of the GCI@nanofilm is rougher compared to lysozyme protein film undergoing phase transition via cysteine (PTLC) (**Figure** **1**C1, D1), attributed to the incorporation of IR783(Figure S1, Supporting Information).^[^ ^19^ ^]^ The water contact angle (WCA) on the same material remains basically unchanged on the PTLC and GCI@nano films (Figure S2, Supporting Information). This indicates that doping with GOx, CAT, and IR783 does not affect the film formation.

The cross‐sectional SEM image of the GCI@nanofilm shows a thickness of ≈675 ± 26.46 nm (Figure 1D2). The β‐sheet‐rich GCI@nanofilm significantly enhances ThT fluorescence specificity, as shown by the confocal microscopy images (Figure 1B1), displaying a light green color with clear outlines. Further, we monitor the kinetics of the reaction system by detecting free thiol groups using N‐(1‐Pyrene) Maleimide (NPM) staining(Figure 1E,F),^[^ ^22^ ^]^ hydrophobic groups using 1‐Anilinonaphthalene‐8‐Sulfonic Acid (ANS) staining, and microenvironment of tryptophan (Trp) groups by their intrinsic fluorescence(Figure 1G).^[^ ^23^ ^]^ These results show that as reaction time increases, the free thiol groups and hydrophobic groups in the system continuously increase, reaching equilibrium after 250 min. Moreover, in this system, cysteine does not alter the structure of the two enzymes, thereby ensuring the retention of their activity. Also, during the phase transition of lysozyme, significant changes occur in the primary secondary structure, transitioning from α‐helix to β‐sheet, as verified by circular dichroism (CD) spectra (Figure 1H) and the deconvolution of the amide I band in Fourier‐transform infrared (FTIR) spectra (Figure S3, Supporting Information).^[^ ^24^ ^]^ Based on our previous studies, X‐ray photoelectron spectroscopy (XPS) elemental analysis of the GCI@nanofilm reveals the exposure of various functional groups on the surface, including C—H/C—C, C—N, C—O, C—S, O=C—N, and O=C—O (Figure S4, Supporting Information). These groups originate from hydrophilic and hydrophobic amino acid residues. These active groups interact with the material surface through coordination bonds, electrostatic interactions, hydrogen bonds, and hydrophobic interactions, effectively promoting the adhesion of the nanofilm to various substrates.

### 2.2 Properties and Performance of the GCI@nanofilm

**Figure 2:**
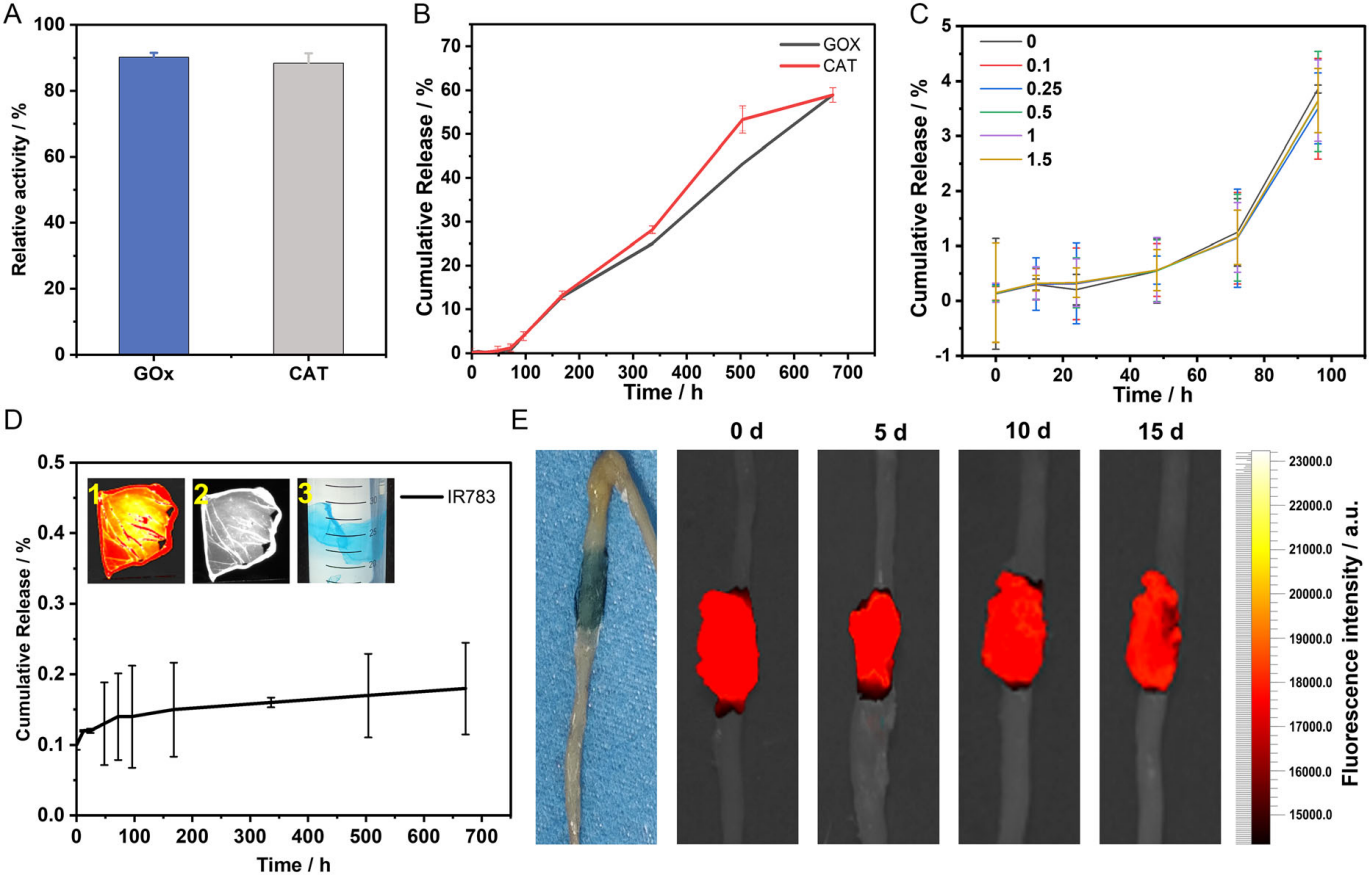
Various performances of GCI@nanofilm. A) Enzymatic activity of GOx and CAT. B) Release profiles of GOx and CAT from GCI@nanofilm in saline over time. C) The total release amount of FITC‐CAT and FITC‐GOx under different power irradiations. D) Release profile of IR783 from GCI@nanofilm in saline over time. D1 represents the chemiluminescence image of GCI@nanofilm captured using an in vivo imaging system, D2 shows the optical image, and D3 shows the optical photograph of GCI@nanofilm after being soaked in physiological saline for 3 months. E) Optical and fluorescence images of simulated intestinal movement in vitro.

The film demonstrated excellent loading capacity, with IR783 loading exceeding 0.17 μg cm^−2^, CAT loading at 7.41 μg cm^−2^, and GOx loading at 20.37 μg cm^−2^. Enzymes encapsulated in the phase‐transition protein film must retain their biocatalytic activity, which is crucial for therapeutic efficacy. We assessed the enzymatic activity of GOx and CAT in the GCI@nanofilm using microenzymatic activity assays. The results showed that the enzymatic activities of GOx and CAT were maintained above 90% (**Figure** **2**A and S5, Supporting Information), highlighting the excellent retention of enzymatic function. Given our treatment approach involves in vivo drug delivery, we further conducted drug release experiments for the GCI@nanofilm. The release of GOx and CAT reached ≈60% in 4 weeks (Figure 2B), with the average daily release of active GOx and CAT per square centimeter being 0.14 and 0.39 μg, respectively. This indicates that GOx and CAT released from the GCI@nanofilm can function effectively during the treatment period, achieving sustained drug delivery. To investigate the effect of light irradiation power on the release of compounds, we used an 808 nm laser to irradiate the GCI@nanofilm grafted with fluorescein isothiocyanate (FITC)‐GOx and CAT and tested the total drug release. The results showed that increasing the laser power from 0.1 to 1.5 W had little effect on the drug release rate compared to the nonirradiated group (Figure 2C). Due to its covalent bonding with lysozyme (Figure S6, Supporting Information), IR783 is hardly released throughout the process (Figure 2D), consistently exhibiting bright signals in an in vivo imaging system (Figure 2D1,D2), and remained unreleased in physiological saline for 3 months (Figure 2D3). Furthermore, it continuously exhibits photodynamic effects under illumination, avoiding metabolic inactivation.

Considering the specific drug delivery site, the adhesion stability and wear resistance of GCI@nanofilm are crucial. We conducted nanoscratch tests and in vitro intestinal motility simulation experiments. Nanoscratch testing is a suitable method for evaluating the adhesion and wear resistance of GCI@nanofilm. The normal force (Fz) and friction coefficient (*μ*) are important indicators of adhesion and wear performance. The experimental results show that the Fz of GCI@nanofilm is 0.635 ± 0.01909 mN. Although this value is not ideal, data from previously published articles by our research group indicate that after 180° peeling, the peel strength between PTL prepared with 7 or 14 mg mL^−1^ lysozyme and glass exceeds 1232 N m^−1^.^[^ ^25^ ^]^ Coupled with subsequent in vitro motion simulation experiments, this sufficiently demonstrates its adhesion to intestinal tissue. The friction coefficient (*μ*) is 0.169 ± 0.0023865, which shows wear resistance comparable to many excellent wear‐resistant films or coating materials. For example, the DLC coating on medical titanium alloy has a friction coefficient of 0.11,^[^ ^26^ ^]^ and the silver‐doped DLC thin film coating with a chromium interlayer (Ag‐DLC/Cr) has a friction coefficient of 0.19 ± 0.04,^[^ ^27^ ^]^ indicating that GCI@nanofilm is fully suitable for applications in the intestine. In the in vitro motility simulation experiment, BALB/c mice were euthanized and colonic tissue was extracted. The GCI@nanofilm was adhered to the isolated intestines of BALB/c mice and then placed in physiological saline at 4 °C (with 1.25% penicillin added), rotating at 100 rpm. Two additional segments of intestines are placed in each beaker to simulate mutual contact and peristalsis of the intestines.^[^ ^28^ ^]^ The adhesion of the GCI@nanofilm is monitored by measuring fluorescence intensity at 0th, 5th, 10th, and 15th day. The results show (Figure 2E and S8, Supporting Information) that the fluorescence signal on the intestines of BALB/c mice almost does not decrease over time (Figure S9, Supporting Information), and the fluorescent area does not significantly diminish. This indicates that the GCI@nanofilm exhibits excellent adhesion and stability on the surface of mouse intestines, fully adapting to intestinal movements.

### 2.3 In Vitro Therapeutic Efficacy of GCI@nanofilm

**Figure 3:**
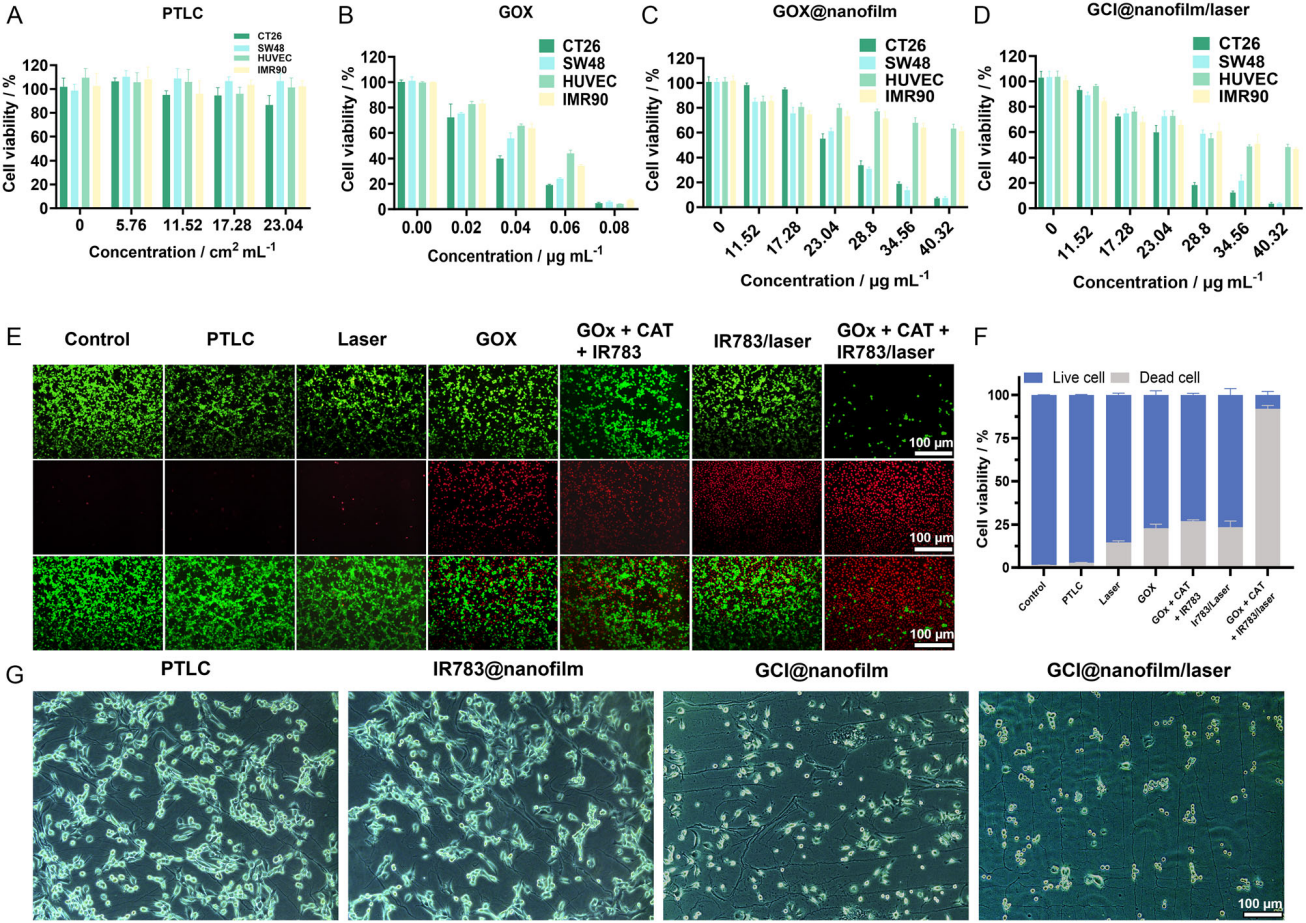
Synergistic antitumor effects of GCI@nanofilm in vitro. A) Viability of CT26.WT, SW48luc, HUVEC, and IMR90 cells after coincubation with increasing concentrations of PTLC extract. B) Viability of CT26.WT, SW48luc, HUVEC, and IMR90 cells after coincubation with free GOx. C) Viability of CT26.WT, SW48luc, HUVEC, and IMR90 cells after coincubation with increasing concentrations of GOx@nanofilm (concentration based on the total amount of GOx in the GOx@nanofilm). D) Viability of CT26.WT, SW48luc, HUVEC, and IMR90 cells after coincubation with GCI@nanofilm (concentration based on the total amount of GOx in the GCI@nanofilm) and exposure to light. E) Live/dead staining images of SW48luc cells after different treatments, scale = 250 μm. F) Proportion of live and dead cells. G) Optical images of cell states on GCI@nanofilm under different treatment conditions, scale =100 μm.

We evaluated the cytotoxicity of PTLC (ranging from 5.76 to 23.04 cm^2^ mL^−1^) on both tumor and normal cells using a CCK‐8 assay. The results showed no significant cytotoxic effects on either cell type (**Figure** **3**A). When cocultured with free GOx (0.02 to 0.08 μg mL^−1^) for 4 h, over 94% of the cells died at the concentration of 0.08 μg mL^−1^, and the tumor cells exhibit a significantly higher mortality rate than normal cells at 0.04 μg mL^−1^ (Figure 3B).^[^ ^29^ ^]^ This phenomenon can be attributed to the Warburg effect, where tumor cells rely more on anaerobic glycolysis for metabolism, leading to increased glucose consumption.^[^ ^30^ ^]^

Next, the cell inhibitory effect of GOx@nanofilm was tested. Based on the drug loading and release rate, it was calculated that after 24 h of coincubation with the cells, the cumulative release amount of GOx in the different gradient treatment groups (depending on the total GOx in the film) was ≈0.02 g, 0.03, 0.04, 0.05, 0.06, and 0.07 μg. Compared to the same concentrations of free GOx, the inhibitory effect of GOx@nanofilm on colon cancer cell lines was only insignificantly reduced, maintaining the basic treatment effect (Figure 3C). The reason for this phenomenon is that GOx is encapsulated in the protein film, which reduces the contact between the enzyme and glucose. Additionally, it is also related to the relatively slow release of the enzyme. The film's sustained release properties allow for long‐term, gradual enzyme release, avoiding the sudden release of the drug that could irritate normal intestinal tissues and the peritoneum. Compared to the GOx@nanofilm, the cell survival rate in the GCI@nanofilm/laser group did not significantly decrease, because the H_2_O_2_ produced by GOx was rapidly decomposed into oxygen by CAT. In an in vitro environment, where hypoxia is not an issue, this did not significantly enhance the cytotoxicity. However, the GCI@nanofilm/laser group still exhibited excellent antitumor effects.^[^ ^31^ ^]^

Next, we assess the survival rates of normal and cancer cell lines treated with GCI@nanofilm (Figure 3D). After coculturing in the dark for 24 h and subsequently irradiating with an 808 nm laser (10 min illumination followed by a 1 h interval and another 10 min illumination), the survival rates of CT26.WT and SW48luc cells treated with 40.32 μg mL^−1^ (24 h cumulative release of 0.07 μg) are below 4%, whereas normal cell lines HUVEC and IMR90 have survival rates above 40%.^[^ ^32^ ^]^ These results indicate that the GCI@nanofilm‐based multienzyme‐involved disulfidptosis and PDT synergistic therapy exhibits significantly higher toxicity to tumor cells, due to the greater sensitivity of tumor cells to glucose deprivation.^[^ ^33^ ^]^

The synergistic therapeutic effect of the GCI@nanofilm is further evaluated using a live/dead cell staining assay. As shown in Figure 3E,F, the cell death rates for free GOx, GOx + CAT + IR783 group, and IR783/laser group on SW48luc cells are 22.88%, 26.91%, and 23.45%, respectively, indicating limited efficacy of single starvation therapy and PDT. However, combining these treatments together with GCI@nanofilm, 91.77% of SW48luc cells are killed, demonstrating a greater cytotoxicity than single treatments, highlighting the synergistic effect of disulfidptosis and PDT combined with starvation therapy.

**Figure 4:**
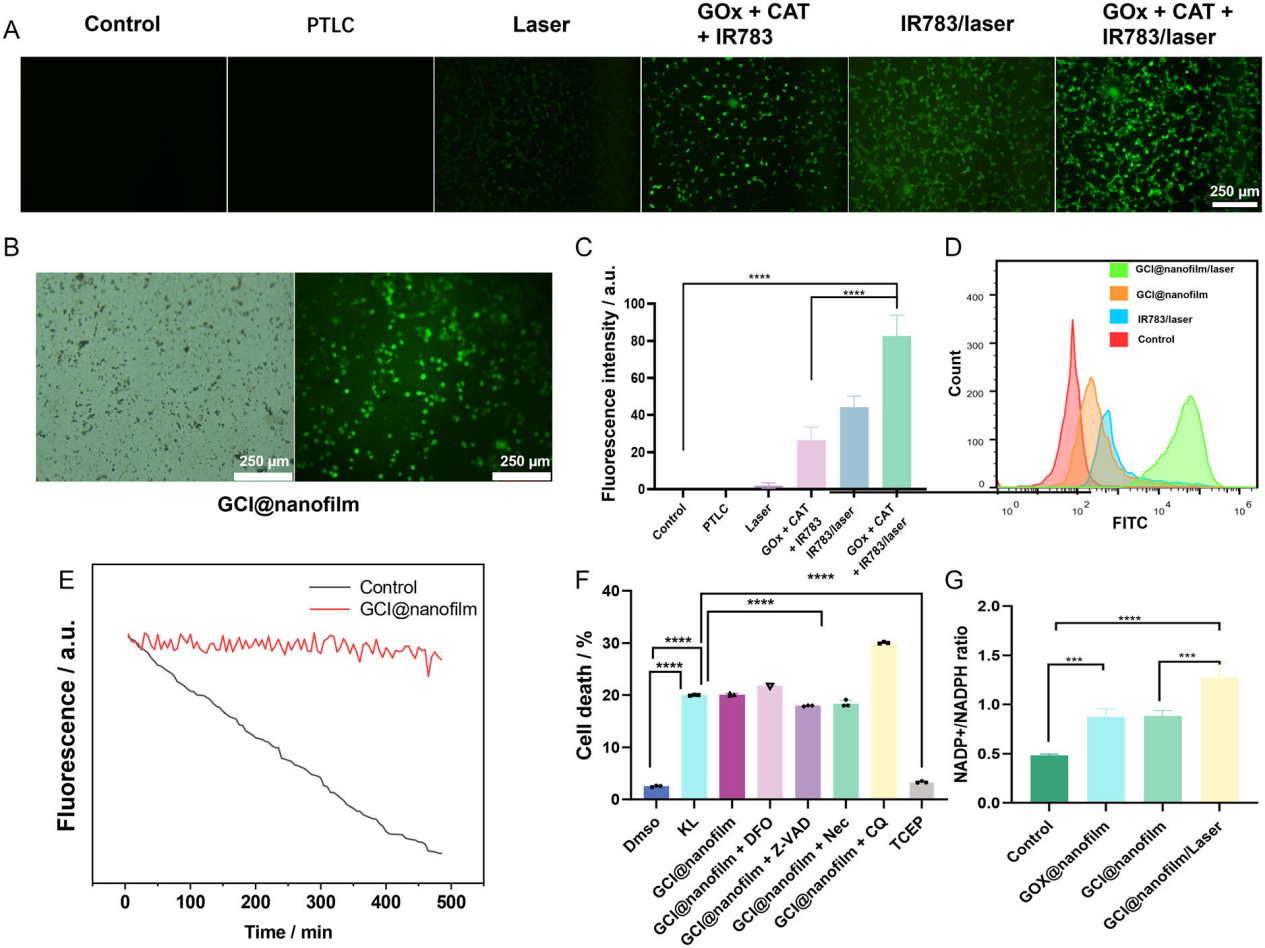
A) Inverted fluorescence microscope images of intracellular ROS generation in SW48luc cells treated with PTLC, laser irradiation, free GOx, CAT, and IR783, free IR783/laser, free GOx, CAT, and IR783/laser. B) ROS images of GCI@nanofilm, scale = 250 μm. C) Quantitative analysis of ROS fluorescence intensity under different treatment conditions. D) Flow cytometry analysis of ROS under different treatment conditions. E) NPM fluorescence intensity over time for control group and GCI@nanofilm, showing intracellular disulfide bond accumulation. F) Viability of SW48luc cells under different treatment conditions, showing specific rescue of cell death induced by GCI@nanofilm with disulfidptosis inhibitor. G) NADP^+^/NADPH ratio under different treatment conditions, showing depletion of NADPH by GCI@nanofilm and GCI@nanofilm/laser. ^∗^ *p* < 0.05, ^∗∗^ *p* < 0.01, ^∗∗∗^ *p* < 0.001.

The generation of intracellular singlet oxygen (^1^O_2_) is assessed using the reactive ROS probe DCFH‐DA. Compared to the control group, the PTLC group, and the single‐illumination group, the ^1^O_2_ levels in the GOx + CAT + IR783 group and the IR783 group are 44.14 and 26.57, respectively, while the GOx + CAT + IR783/laser group exhibits ^1^O_2_ levels of 82.62 (**Figure** **4**A,C). The differences in fluorescence intensity levels in cancer cells can be attributed to the generation of H_2_O_2_ by GOx and the production of ROS during PDT. Due to the sustained release effect of the film, we coculture the film with cells for 72 h, and as shown in Figure 4B, the film also successfully generates ROS, initiating PDT. Consistent with the ROS probe results, flow cytometry experiments in the control, GOx + CAT + IR783, IR783/laser, and GOx + CAT + IR783/laser groups show results consistent with those obtained from the ROS probe (Figure 4D).^[^ ^34^ ^]^ These results confirm the successful initiation of PDT at the cellular level.

To demonstrate the occurrence of disulfidptosis, we first conduct fluorescent probe staining using NPM to detect the reduction of free thiols in cells, reflecting the accumulation of disulfide bonds. As shown in Figure 4E, the fluorescence intensity in the GCI@nanofilm group decreases while remaining unchanged in the control group. To confirm that the GCI@nanofilm indeed induces disulfidptosis, we next used inhibitors of cell death pathways, including 1 mmol L^−1^ TCEP (disulfidptosis inhibitor), 80 μmol L^−1^ DFO (ferroptosis inhibitor), 10 μmol L^−1^ Z‐VAD (apoptosis inhibitor), 5 μmol L^−1^ Nec (necroptosis inhibitor), and 25 μmol L^−1^ CQ (autophagy inhibitor), to test which manners work (Figure 4F).^[^ ^35^ ^]^ Only adding TCEP effectively inhibits the cell death, further confirming the occurrence of disulfidptosis induced by GCI@nanofilm. The key trigger for disulfidptosis is the depletion of intracellular NADPH. We measure the NADP^+^/NADPH levels and find that both the GOx@nanofilm group and the GCI@nanofilm group cause a decrease in NADP^+^/NADPH levels, and the GCI@nanofilm/laser group further depletes NADPH, likely due to the disruption of cellular redox homeostasis by ROS generated during PDT. In summary, we demonstrate that GCI@nanofilm induces an increase in cellular disulfide bonds, excludes other cell death pathways, and further confirms the decrease in NADPH levels, thus confirming its induction of disulfidptosis.

### 2.4 In Vivo Stability and Degradation

**Figure 5:**
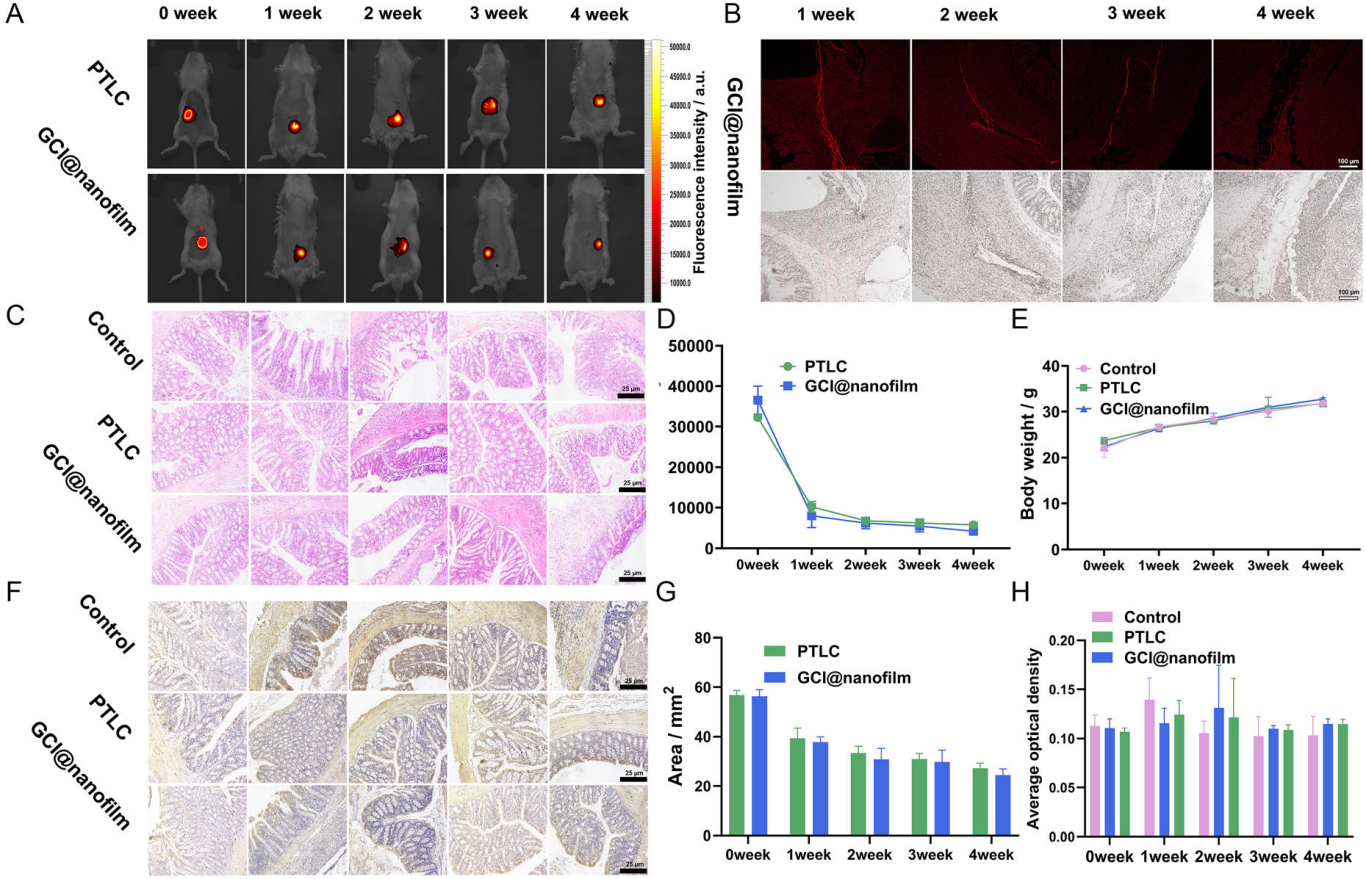
A) In vivo imaging of IR783‐grafted PTLC film and GCI@nanofilm at 0th, 1th, 2th, 3th, and 4th week. B) Confocal images showing the degradation of GCI@nanofilm in the intestine, scale = 100 μm. C) H&E staining images of intestinal tissues in the control (sham surgery group), PTLC‐adhered, and GCI@nanofilm‐adhered groups at 0th, 1th, 2th, 3th, and 4th week, scale = 25 μm. D) Quantitative analysis of fluorescence intensity in in vivo imaging. E) Body weight of mice in the control, PTLC, and GCI@nanofilm groups over time. F) IL‐6 images of mice in the control, PTLC, and GCI@nanofilm groups at 0th, 1th, 2th, 3th, and 4th week, scale = 25 μm. G) Fluorescence area of PTLC and GCI@nanofilm groups over time. H) AOD values of IL‐6 in mice from the control, PTLC, and GCI@nanofilm groups over time.

Before conducting in vivo experiments, we performed a hemolysis test to preliminarily assess whether the GCI@nanofilm and PTLC are suitable for in vivo testing (Figure S10, Supporting Information). The results showed no hemolysis at concentrations up to 34.56 cm^2^ mL^−1^ for GCI@nanofilm and no hemolysis for PTLC either, indicating high biocompatibility of the nanofilm material.^[^ ^36^ ^]^ In the in vivo test, the GCI@nanofilm and IR783‐labeled PTLC were applied to the surface of the colon in anesthetized BALB/c mice (Figure S11, Supporting Information). The results indicated that both PTLC and GCI@nanofilm remained stable around colon for over 4 weeks (**Figure** **5**A).^[^ ^37^ ^]^ When the duration was extended to the 6th week, the fluorescence of GCI@nanofilm had nearly disappeared, indicating that GCI@nanofilm can provide long‐term sustained drug release, perfectly fitting the therapeutic scenario for inhibiting CRC recurrence (Figure S12, Supporting Information). The same degradation trend was also observed using confocal microscopy (Figure 5B and S13, Supporting Information). To evaluate the impact on surrounding tissues, we conducted hematoxylin and eosin (H&E) staining, and the results showed no adverse effects on tissue morphology (Figure 5C). Quantitative analysis of fluorescence intensity showed significant degradation within the first week, followed by a stable trend from the second to the 4th week (Figure 5D). Monitoring the body weight of the mice showed steady growth (Figure 5E), and IL‐6 staining results showed that compared to the control group, PTLC and GCI@nanofilm did not cause significant inflammation in intestinal tissues (Figure 5F) and the area slowly decreased over time, which is related to the macrophage response (Figure 5G).^[^ ^38^ ^]^ Quantitative analysis of IL‐6 levels using ImageJ showed that the average optical density (AOD) values for the PTLC and GCI@nanofilm groups were within the normal range, with no significant difference compared to the control group.^[^ ^39^ ^]^ Overall, PTLC and GCI@nanofilm remained stable in the colon for at least 4 weeks without adverse effects on normal intestinal tissues (Figure 5H).

### 2.5 In Vivo Antitumor Efficacy of GCI@nanofilm Under Laser Irradiation

**Figure 6:**
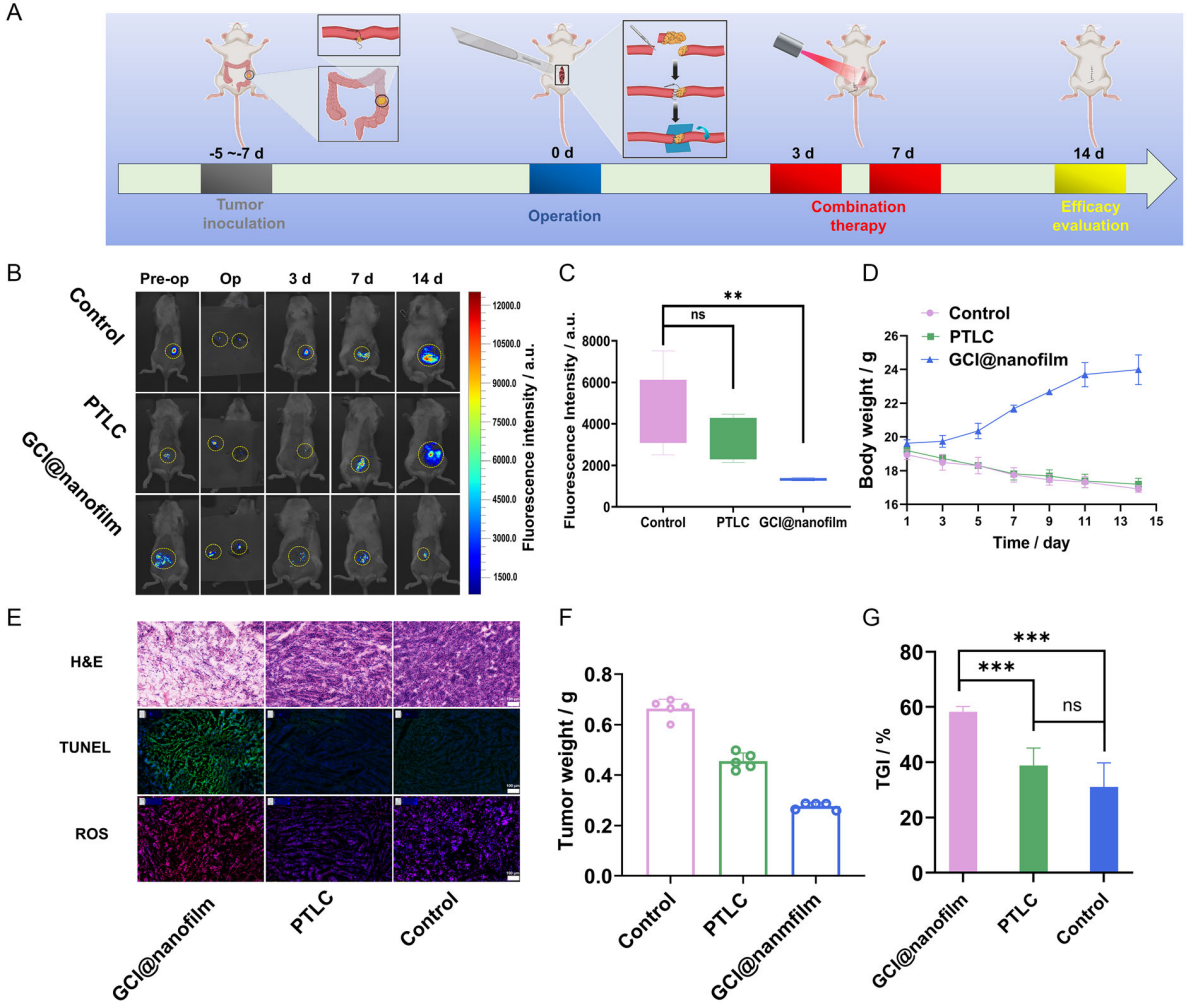
A) Schematic of the modeling and treatment process for the incomplete resection of orthotopic CRC. B) In vivo tumor imaging of the control group (no film), PTLC group, and GCI@nanofilm group before surgery, during surgery (90% of the tumor tissue removed, indicated by the yellow circle on the left), and at 3th, 7th, and 14th day postsurgery. C) Quantitative analysis of in vivo tumor fluorescence intensity in the control, PTLC, and GCI@nanofilm groups after 14 days of treatment. D) Body weight of mice in the control, PTLC, and GCI@nanofilm groups over time. E) H&E, TUNEL, and ROS images of tumor tissues in the control, PTLC, and GCI@nanofilm groups, scale = 100 μm. F) Tumor weight in the control, PTLC, and GCI@nanofilm groups after 14 days of intervention. G) Tumor inhibition rate in the control, PTLC, and GCI@nanofilm groups after 14 days of intervention, showing significant tumor inhibition by the GCI@nanofilm group compared to the control and PTLC groups. ^∗^ *p* < 0.05, ^∗∗^ *p* < 0.01, ^∗∗∗^ *p* < 0.001.

To more accurately simulate clinical conditions, the tumor microenvironment, and the recurrence of residual cancer tissue, we established an incomplete resection orthotopic CRC model. The in vivo antitumor efficacy of GCI@nanofilm under laser irradiation was evaluated. **Figure** **6**A illustrates the entire surgical and intervention process. The SW48luc tumor previously implanted subcutaneously in BALB/c‐nu/nu mose was excised and cut into small fragments (1 mm^3^) and then sutured onto the colonic wall of NOD SCID mice. After ≈5–7 days, intraperitoneal bioluminescent signals could be observed, indicating that the model had been successfully established. 15 mice successfully modeled with orthotopic CRC were selected to establish an orthotopic CRC incomplete resection model. The mice were randomly divided into three groups (five mice per group). In the orthotopic CRC model, 90% of the tumor was excised, followed by intestinal anastomosis, and the remaining 10% of the tumor was completely covered with GCI@nanofilm and PTLC. Laser irradiation was performed on days 3 and 7 postsurgery. Tumor signals were monitored on day 3th, 7th, and 14th (Figure 6B).^[^ ^40^ ^]^ The results showed that the tumor fluorescence intensity in the GCI@nanofilm/laser group significantly decreased, with a growth inhibition rate of 58.24%. In contrast, the other two groups exhibited noticeable tumor recurrence (Figure 6G), demonstrating the excellent antitumor effect of the GCI@nanofilm/laser group.

After 14 days of treatment, mice were sacrificed, and tumor tissues were extracted for H&E staining, terminal deoxynucleotidyl transferase dUTP nick end labeling (TUNEL) staining, and reactive ROS staining. These analyses confirmed that the GCI@nanofilm group had the most severe tumor damage (Figure 6E).^[^ ^41^ ^]^ H&E staining showed the loss of normal tumor tissue structure in the GCI@nanofilm group, and cancerous tissue infiltration in the colon in the control and PTLC groups (Figure S14, Supporting Information); TUNEL staining indicated the highest number of apoptotic cells in the experimental group; ROS cryosections showed significantly higher ROS in the experimental group compared to the control group. Additionally, during this period, the average body weight changes in the control and PTLC groups were greater than in the GCI@nanofilm group. The GCI@nanofilm group began to show weight gain 1 week postsurgery, indicating a strong antitumor effect in the orthotopic CRC recurrence model. Since this experiment used a complex orthotopic CRC incomplete resection model, it more accurately reflects the real microenvironment of CRC.^[^ ^42^ ^]^ After 14 days postsurgery, two mice in the control group (out of 5 per group) developed liver metastases (Figure S15, Supporting Information), while no distant metastases were observed in the GCI@nanofilm group, suggesting that GCI@nanofilm might also have a metastasis‐inhibiting effect.^[^ ^43^ ^]^

## 3 Discussion

Local therapy has shown a great potential in inhibiting the recurrence of residual cancer tissue at the surgical site following surgery for advanced CRC. Compared to traditional systemic administration, which often has significant side effects, strategies of direct treatment at the lesion site offer clear advantages for resectable CRC, such as rapid intraoperative deployment, increased local drug concentration, and reduced systemic toxicity.^[^ ^44^ ^]^ The effectiveness of local therapy is highly dependent on the delivery material. The colon's large and complex range of motion, coupled with the moist environment of the intestinal wall and abdominal cavity, makes materials prone to detachment. Therefore, only highly adhesive materials can ensure that the drug remains at the intended site long enough to be effective. The 2D amyloid protein film prepared in this study has many advantages that effectively address the aforementioned issues. Compared to other materials, the prepared protein film is thinner, exhibits stronger wet adhesion, possesses an ultrahigh enzyme activity retention capability, offers stable drug release, and demonstrates excellent biocompatibility.

The GCI@nanofilm, with a thickness of up to 600 nm, offers enhanced flexibility and a larger specific surface area. This allows it to fit seamlessly onto the uneven surfaces of the colon and maximize the effects of the loaded active molecules, outperforming traditional micrometer‐ or millimeter‐sized injectable hydrogels and electrospun materials. More importantly, the nanofilm's amyloid‐like structure, confirmed by CD, FITR, and ThT staining, provides a structural basis for its biomimetic wet adhesion, similar to barnacles and bacterial biofilms. The functional groups on the surface of GCI@nanofilm, including carboxyl, hydroxyl, and amino groups, facilitate multiscale and multisite adhesion, ensuring outstanding capability on wet surfaces.^[^ ^16^ ^]^ Besides, the film exhibits Janus surface properties, with one side being adhesive and the other nonadhesive.^[^ ^45^ ^]^ This ensures drug efficacy while minimizing the impact on normal intestinal motion, significantly improving the quality of life for patients. Benefiting from the aforementioned advantages, in the nanoscratch test, GCI@nanofilm exhibited a binding force of 0.635 al intestinal motion, signifi friction coefficient of 0.19 ± 0.04. In an in vitro experiment simulating intestinal movement at 100 rpm, the film remained intact and adhered firmly over a period of 15 days, demonstrating superior wet surface adhesion and flexibility. Unlike other materials that typically require additional adhesive molecules, such as adding carbomer powder in electrospinning or polydopamine in hydrogels, the adhesion of this protein film originates from its own structure,^[^ ^21^ ^]^ making the adhesion performance more stable and synthesis simpler.

The entire reaction process is highly specific, with cysteine reacting only with lysozyme, which is a crucial basis for retaining the activities of GOx and CAT. The film formed through the reduction reaction can retain 90% of GOx and CAT activities. In our in vitro release experiments, it was found that there was a sustained and stable release over 4 weeks, and the dose of the released enzymes could ensure effective treatment, which is beneficial for prolonging the drug action time and significantly superior to the release performance of hydrogels and other materials. Mass spectrometry (MS) confirms the covalent binding of IR783 with lysozyme in the film. In vitro release experiments reveal that the GCI@nanofilm exhibits negligible release of IR783 in physiological saline over a 30 day period. Upon 808 nm laser irradiation, local reactive ROS generation is sustained without concerns regarding reduced efficacy due to decreased drug concentration. For materials used in localized drug delivery, biocompatibility and degradation are particularly crucial due to direct contact with tissues and surgical incisions. Consequently, we conducted in vivo biocompatibility and stability tests by in situ attachment of the GCI@nanofilm to the colorectal region of BALB/c mice. Samples were collected at 0th, 1th, 2th, 3th, and 4th week and analyzed for biocompatibility and inflammatory response through HE staining and IL‐6 assays. The results indicated that the protein film material did not induce significant inflammatory responses in vivo. H&E staining showed that the intestinal tissue maintained normal morphology, and the AOD values of IL‐6 remained within the normal range. Despite the protein‐based nature of the material, which typically elicits local inflammatory responses, the experimental results demonstrated no significant inflammation. Since the protein film did not degrade within 4 weeks, we extended the timeline to investigate the degradation period. We found that the fluorescence of IR783 nearly disappeared by the 6th week, ensuring prolonged stability for in vivo drug delivery. Overall, this sustained release and prolonged degradation time can ensure spatiotemporal matching treatment during the recurrence process of CRC surgery.

After successfully preparing the GCI@nanofilm, we designed and established a more complex orthotopic CRC incomplete resection model to evaluate its therapeutic efficacy, aiming for a more realistic clinical application scenario. Compared to the subcutaneous CRC models commonly used in most studies, the orthotopic CRC incomplete resection model effectively replicates the natural growth environment of tumors within the colorectal region. This model includes the interactions between the tumor and surrounding tissues, blood vessels, and the microenvironment, thereby more accurately simulating residual tumor postsurgery and local recurrence. This provides a more clinically relevant basis for evaluating postoperative recurrence prevention strategies and reasonably assesses the efficacy and bioavailability of the localized drug delivery system. Experimental results indicated that within 14 days postsurgery, 40% of the mice in the control group showed liver metastasis, which occurs significantly faster than the distant metastasis observed over months in subcutaneous models. The GCI@nanofilm demonstrated excellent therapeutic efficacy in in vivo experiments, achieving a tumor inhibition rate of 58.24%. This efficacy is attributed to the synergistic application of disulfidptosis and PDT.

Disulfidptosis, discovered in 2023, is a novel cell death mechanism that regulates abnormal tumor metabolism, offering broad prospects for cancer treatment. This type of cell death depends on intracellular glucose deprivation and can be triggered by glucose transporter inhibitors or related metabolic enzymes. In contrast, GOx, due to its high specificity for glucose, can effectively deplete glucose at the tumor site in local treatment applications, successfully cutting off the tumor's glucose supply. This makes it more efficient and less toxic compared to glucose transporter inhibitors. In vitro experiments, we detected an increase in intracellular disulfide bonds and measured the NADP^+^/NADPH ratio under different treatment conditions. We found that the decline in NADPH levels in the GCI@nanofilm/laser treatment group was significantly greater than that in the GCI@nanofilm group, likely due to the consumption of NADPH by reactive ROS generated during PDT. The disulfidptosis inhibitor TCEP effectively inhibited GOx‐induced cell death, while inhibitors of other cell death pathways had no effect, thereby fully demonstrating that the localized treatment strategy designed in this study can effectively induce disulfidptosis in colon cancer cells through GOx. Live/dead cell staining and CCK‐8 assays showed that GOx exerted a certain cytotoxic effect. Laser‐induced PDT also exhibited some efficacy. However, the effects of both treatments alone were not significant, with the inhibition rate of disulfidptosis treatment alone at 26.91% and that of PDT alone at 23.45%. To effectively synergize with PDT, this study further incorporated CAT on the basis of GOx, which catalyzed the conversion of hydrogen peroxide produced during disulfidptosis into oxygen. Oxygen not only further promotes disulfidptosis but also alleviates the hypoxia of tumor tissues, thus enhancing PDT efficacy. Synergy testing results revealed that the combined use of these two methods exhibited a significant synergistic effect, achieving a cell inhibition rate of 91.77%. In the in vivo orthotopic CRC incomplete resection model, this combination also effectively inhibited tumor recurrence. This synergistic effect is also attributed to the foundational properties provided by the materials.

## 4 Conclusion

This study addresses the clinical challenge of postoperative recurrence of CRC by developing a localized 2D protein nanofilm treatment platform. Utilizing amyloid‐like film technology, it combines disulfidptosis and PDT for highly efficient synergistic treatment. The protein nanofilm boasts a simple and gentle preparation process, excellent structural stability and biocompatibility, high enzymatic activity, strong and stable wet adhesion, suitable degradability, and sustained drug release. These features enable the integration of enzyme cascade‐activated disulfidptosis and PDT into a single system, offering localized and spatiotemporal synergistic treatment for postoperative recurrence of CRC. In an animal model simulating clinical surgery, applying the GCI@nanofilm to the postoperative colonic site resulted in nearly 60% tumor inhibition after 14 days of treatment. This study provided innovative ideas and methods for CRC treatment, promoting the development of personalized medicine and precision therapy and offering more effective treatment options.

## 5 Experimental Section

5.1

5.1.1

##### Chemical and Materials

Lysozyme, GOx (S10021), and CAT (S10037) were purchased from Yuanye Biotechnology Co., Ltd. The GOx activity assay kit (BC0695), catalase activity assay kit (BC0205), and DCFHDA ROS probe kit (D6470) were purchased from Solarbio. L‐cysteine (C108237), tris (2‐carboxyethyl) phosphine (T107252), and IR783 (I157644) were purchased from Aladdin Biochemical Technology Co., Ltd. The NADP^+^/NADPH assay kit (S0176) was purchased from Beyotime Biotechnology. The CCK‐8 assay kit (G4103‐5ML), live/dead cell staining kit (G1707‐100T), and interleukin‐6 (IL‐6) detection kit (GB300007‐*M*‐10μg) were purchased from Servicebio Biotechnology Co., Ltd. All other chemical reagents were purchased from Sinopharm Group. The SW48luc and CT26.WT cell lines were obtained from ATCC. BALB/c mice and BALB/c‐nu/nu mice were purchased from Beijing HFK Bioscience Co., Ltd. NOD SCID mice were purchased from SPF (Beijing) Biotechnology Co., Ltd. Unless otherwise stated, all chemicals and reagents were of analytical grade and used according to the supplied standards.

##### Preparation of GCI@nanofilm

Preparation of Solution 1: Cysteine buffer solution (20 mg mL^−1^, pH = 10); Solution 2: Lysozyme buffer solution (20 mg mL^−1^). 10 mg of GOx, 10 mg of CAT, and 10 mg of IR783 were added to 5 mL of Lysozyme buffer solution (20 mg mL^−1^). Subsequently, 5 mL of cysteine buffer solution (20 mg mL^−1^, pH = 10) was added to the mixture in a 1:1 volumetric ratio. 600 μL of the resulting solution was then dropped onto each 24 × 24 mm glass slide, followed by incubation at 37 °C for 10 h. Finally, the glass slides were placed in ultrapure water, and the phase transition lysozyme nanofilm formed at the air–liquid interface was collected for further use (Figure 1A).

##### Characterization and Method: Scanning Electron Microscopy Measurements

Field‐emission SEM observations were conducted on SU8020 (Hitachi). The PTLC and GCI@nanofilm samples were put on the electrically conductive adhesives and spray gold. The cross‐section samples of GCI@nanofilm were wetting‐off in liquid nitrogen.

##### X‐ray Photoelectron Spectroscopy Measurements

XPS of the PTLC and GCI@nanofilm samples was performed by an AXIS ULTRA (Kratos Analytical Ltd.).

##### WCA Measurement

The WCA of PTLC and GCI@nanofilm was measured on OCA 20 (Data Physics, Germany).

##### Fourier‐Transform Infrared Spectroscopy Measurements

FTIR spectra were obtained using a Vertex 70 V spectrometer (Bruker Inc., Germany). FTIR spectra were obtained between 400 and 4000 cm^−1^ with a resolution of 1 cm^−1^ using an Alpha‐T spectrometer (Bruker) in the KBr disk method.

##### Laser Scanning Confocal Microscopy Measurements

Thioflavin T (ThT)‐stained GCI@nanofilm were tested using an Olympus laser scanning confocal microscope FV 1200 apparatus. The activator was 405 nm.

##### Circular Dichroism Spectrum Measurement

Far‐UV CD spectra were collected under a constant nitrogen flush at 25 °C and recorded with 2.0 nm from 200 to 260 nm.

Monitoring the Changes in Protein Hydrophobic Regions Using ANS Fluorescence Probe. In a 96‐well plate, 20 μL of an ANS (8‐Anilino‐1‐naphthalenesulfonic acid, 200 μm) solution was mixed with 90 μL of different protein solutions, including lysozyme (20 mg mL^−1^), GOx (1 mg mL^−1^), and CAT (1 mg mL^−1^). The mixed solutions were then placed in a microplate reader, and 90 μL of a cysteine (20 mg mL^−1^) solution was added. The fluorescence intensity of the samples was recorded over time using an excitation wavelength of 355 nm and an emission wavelength of 470 nm. This method utilized the selective binding of the ANS fluorescent probe to protein hydrophobic regions, allowing for the monitoring of conformational changes of the proteins upon addition of cysteine. The time‐dependent changes in the fluorescence signal can provide insights into the structural dynamics of the proteins.

##### Tryptophan (Trp) Fluorescence Monitoring

90 μL of a cysteine solution (20 mg mL^−1^, pH = 10) was separately mixed with 90 μL of different protein solutions, including lysozyme (20 mg mL^−1^), GOx (1 mg mL^−1^), and CAT (1 mg mL^−1^). The fluorescence intensity of the samples was recorded over time using a Spark multimode microplate (TECAN, Switzerland) reader until the fluorescence intensity remained unchanged. The excitation wavelength was set at 285 nm, and the emission wavelength was set at 340 nm.

Monitoring Changes in Protein Sulfhydryl Groups Using NPM Fluorescence Labeling. In a 96‐well plate, 20 μL of an N‐(1‐pyrenyl) maleimide (NPM, 10 mmol L^−1^ in DMF) solution was separately mixed with 90 μL of different protein solutions, including lysozyme (20 mg mL^−1^), GOx (1 mg mL^−1^), and CAT (1 mg mL^−1^). Then, 90 μL of a cysteine solution (20 mg mL^−1^, pH = 10) was added to the mixtures. The fluorescence intensity of the samples was recorded over time using a microplate reader. The excitation wavelength was set at 330 nm, and the emission wavelength was set at 380 nm.

##### MALDI‐TOF‐MS Characterization of Covalent Conjugation

A lysozyme solution (20 mg mL^−1^) was mixed in equal volume with an IR783 solution (2 mg mL^−1^), and the resulting reaction solution was diluted 10‐fold. 1 μL of the diluted mixture was then combined with an equal volume of α‐cyano‐4‐hydroxycinnamic acid solution (10 mg mL^−1^ in tetrahydrofuran) and thoroughly mixed. The mixture was then spotted on the matrix‐assisted laser desorption/ionization (MALDI) target plate, dried, and subjected to MALDI time‐of‐flight MS analysis to detect changes in the molecular mass of lysozyme before and after the conjugation reaction.

The GCI@nanofilm protein film was placed inside an in vivo imaging system (Smart‐LF, South Korea), and the fluorescence of the material was imaged using the ir783 catalase glucose oxidase imaging mode. This method utilized an in vivo imaging system to detect the fluorescence signal emitted by the GCI@nanofilm protein film.

##### X‐Ray Photoelectron Spectroscopy Measurements

XPS of the PTLC and GCI@nanofilm samples was performed by an AXIS ULTRA (Kratos Analytical Ltd.).

Determination of Drug Encapsulation Efficiency of Sustained‐Release Coating. To ensure complete dissolution of the drug, the prepared GCI@nanofilm was immersed in a 1% ascorbic acid solution and stirred vigorously at room temperature (700 rpm) for 4 h. The resulting solution was then centrifuged (10 000 rpm, 10 min), and the supernatant was collected. The absorbance of the supernatant was measured using a UV spectrophotometer to determine the encapsulated content of IR783, GOx, and CAT in the drug delivery system.

The encapsulation efficiency (EE) was calculated using the following formula.

(1) $$\text{EE}=\frac{W}{W_{0}}\times 100\%$$

where *W*_0_ represents the initial amount of drug loaded, and *W* is the actual amount of drug stored in the sustained‐release coating.

##### Drug Release

Using FITC‐grafted CAT and GOx, we established a standard curve. The GCI@nanofilm was soaked in 100 mL of phosphate buffer (PBS) at 37 °C. Liquid samples were collected at fixed time points. By applying light powers of 0, 0.1, 0.25, 0.5, 1, and 1.5 W to the GCI@nanofilm, we collected 0.5 mL of PBS at time points of 0, 12, 24, 48, 72, and 96 h and then replenished the total liquid volume to 2 mL. After collecting the samples, we measured the absorbance at 450 nm using a UV–vis spectrophotometer to calculate the release amount. Based on these experiments, we investigated the effect of light irradiation power on drug release.5.3. Testing of Mechanical Properties.

##### Nanoscratch Test

The nanoscratch test was conducted to evaluate the mechanical properties of the GCI@nanofilm using a nanoscratch tester (NTS3, Anton Paar). The scratch test was performed by applying a progressively increasing load, starting from zero, at a constant rate. During the test, the tangential force (Fx) and normal force (Fz) were continuously recorded. Each test was repeated three times to ensure reproducibility, and the average values were reported.

(2) $$\mu =\frac{\text{Fx}}{\text{Fz}}$$

##### Adhesive and Stability Testing of GCI@nanofilm

The GCI@nanofilm was attached to the colon of BALB/c mice. An 8 cm segment of the colon containing the material was excised and placed in 4 °C PBS buffer. Samples were collected at 0th, 5th, 10th, and 15th day postimplantation. At each time point, the colon tissue was placed in an in vivo imaging system, and the fluorescence intensity was quantitatively measured. BALB/c mice, *n* = 3.

##### Cell Experiments: Cell Culture

In subsequent experiments involved the in vitro culture of human colorectal adenocarcinoma cells SW48luc and murine CRC cells CT26.WT. SW48luc cells were purchased from WanWu Biotechnology Co (Hefei) and CT26.WT‐luc‐gfp‐puro (WN‐20 062) cells were purchased from Warner Bio (Wuhan) Co.,Ltd. The SW48luc and CT26.WT cells were incubated in high‐glucose liquid culture medium (DMEM, Cytiva, USA) with 10% fetal bovine serum (FBS, Evergreen, China) and 1% v/v antimicrobial of penicillin/streptomycin (MACKLIN) at 37 °C in a humidified atmosphere of 5% CO_2_. During cell culture, the PTLC and GCI@nanofilm used were generated between cells, and the solutions needed were filtered through a sterile filter.

##### Cell Death and Viability Assays

The effects of different materials on CT26.WT, SW48luc, HUVEC, and IMR90 cells were analyzed using a CCK‐8 assay kit (Servicebio). The four types of cells mentioned above were seeded onto a 96‐well plate at a density of 1 × 10^4^ cells and cocultured for 24 h. Subsequently, the optical density (OD) of the culture medium was measured at 450 nm using an automatic microplate reader (Tecan Spark, Switzerland). Cell viability was determined as follows: cell viability (%) = (absorbance of tested compound – absorbance of blank) ÷(absorbance of control – absorbance of blank) × 100.

##### Measurement of ROS

A 48‐well plate was seeded with 6 × 10^4^ cells per well and coincubated with different materials overnight. The cells were then exposed to 808 nm laser irradiation (0.5 W cm^−2^) with interruptions of 5 min between two 10 min irradiation periods, totaling 20 min of exposure. Images were captured using an inverted fluorescence microscope. Quantitative analysis of the images was performed using ImageJ software. Additionally, flow cytometry was used to analyze the cells.

##### Live/Dead Cell Staining Experiment

A 48‐well plate was seeded with 6 × 10^4 ^cells per well and treated with different materials. Subsequently, a live/dead cell staining analysis using Calcein‐AM and propidium iodide (PI) was conducted to investigate the synergistic effects. Images were captured using an inverted fluorescence microscope. Quantitative analysis of the images was performed using ImageJ software.

##### NADP^+^ and NADPH Measurements

Using the NADP^+^/NADPH Assay Kit (Beyotime), total NADP^+^/NADPH levels were first measured according to the instructions, followed by the separate quantification of NADPH. Utilizing the obtained total amounts of NADP^+^ and NADPH from the first two steps, the quantity of NADP^+^ in the sample, as well as the ratio of NADP^+^/NADPH, can be determined.

##### Hemolysis Assay

The experiment included a negative control (physiological saline), two positive controls (distilled water and 0.1% Triton X‐100), PTLC, and GCI@nanofilm, with each group having three parallel experiments. After coincubation with 2% sheep red blood cells for 4 h, the supernatant was collected by centrifugation (10 000 rpm, 2 min) and added to a 96‐well plate. The absorbance at 542 nm was measured using a microplate reader.

##### Animal Experiments: Establishment of Orthotopic Residual Colon Cancer Tissue Model

The SW48luc cells were subcutaneously inoculated at a concentration of 3 × 10^6 ^cells mL^−1^ into the right hip flank of BALB/c‐nu/nu mouse. Once the subcutaneous tumor reached a volume of 50 mm^3^, colonic transplantation was performed. For the transplantation procedure, NOD SCID mice were anesthetized and the abdomen was sterilized with iodine and alcohol swabs. A small midline incision was made, and the colorectal part of the intestine was exteriorized. The serosa at the site where the tumor fragments were to be implanted was removed. Tumor fragments of 1 mm^3^ in size were implanted onto the intestinal wall. An 8‐0 surgical suture was used to penetrate these small tumor fragments and secure them to the intestinal wall. The intestine was then returned to the abdominal cavity, and the abdominal wall was closed with 7‐0 surgical sutures. The animals were kept in a sterile environment. Mice with successfully engrafted tumors underwent 90% tumor resection, leaving a 10% residual tumor, followed by intestinal anastomosis. The model mice were divided into three groups, each consisting of 5 mice: control, PTLC, GCI@nanofilm. The control group received no additional treatment, while the PTLC and GCI@nanofilm groups had their respective PTLC and GCI@nanofilm adhered and completely covered on the residual tumor tissue. 6‐0 sutures were used for the rectus abdominis muscle and 4‐0 sutures for the abdominal wall, establishing models of orthotopic CRC and postresection residual tumor.

##### In Vivo Degradation and Stability

IR783‐labeled PTLC and GCI@nanofilm were adhered to the colon tissue of mice, and quantitative measurements of fluorescence intensity in vivo were conducted using an in vivo imaging system (VISQUE InVivo Smart‐LF) at 0, 1th, 2th, 3th, and 4th week time points. Subsequently, the IR783‐labeled PTLC and GCI@nanofilm, along with surrounding tissues, were harvested for histological examination, including H&E staining and IL‐6 analysis. Confocal microscopy was utilized to capture images of the tissues to observe the degradation of IR783‐labeled PTLC and GCI@nanofilm.

##### Statistical Analysis

All quantitative data represented the mean ± SD. Statistical difference was tested by student's *t*‐test or one‐way analysis of variance using GraphPad Prism 8.0 (GraphPad Software). The statistical significance was assigned at ^*^ *p* < 0.05, ^**^ *p* < 0.01, ^***^ *p* < 0.001, ^****^ *p* < 0.0001.

## Conflict of Interest

The authors declare no conflict of interest.

## Author Contributions

**Man Zhang**: Data curation (lead); Formal analysis (lead); Methodology (lead); Visualization (lead); Writing—original draft (lead). **Ke Li**: Funding acquisition (equal); Investigation (lead); Project administration (lead); Supervision (lead); Validation (lead); Writing—review & editing (lead). **Junhao Kou**: Data curation (lead); Formal analysis (equal); Validation (equal). **Guozhi Lu**: Data curation (equal); Formal analysis (equal); Methodology (lead). **Ling Qiu**: Project administration (lead); Writing—review & editing (supporting). **Chunzhao Yang**: Conceptualization (supporting); Supervision (equal); Writing—original draft (supporting). **Yongchun Liu**: Funding acquisition (supporting); Supervision (lead); Writing—original draft (supporting); Writing—review & editing (lead). **Qi Xue**: Funding acquisition (lead); Supervision (lead); Writing—review & editing (lead). **Peng Yang**: Funding acquisition (lead); Supervision (lead); Writing—review & editing (lead). **Man Zhang** and **Ke Li** contributed equally to this work.

## Supporting information

Supplementary Material

## Data Availability

The data that support the findings of this study are available from the corresponding author upon reasonable request.

## References

[1] R. L. Siegel , K. D. Miller , H. E. Fuchs , A. Jemal , CA‐Cancer J. Clin. 2022, 72, 7. 35020204 10.3322/caac.21708

[2] E. Dekker , P. J. Tanis , J. L. A. Vleugels , P. M. Kasi , M. B. Wallace , Lancet 2019, 394, 1467. 31631858 10.1016/S0140-6736(19)32319-0

[3] H. Brenner , M. Kloor , C. P. Pox , Lancet 2014, 383, 1490. 24225001 10.1016/S0140-6736(13)61649-9

[4] L. H. Biller , D. Schrag , JAMA, J. Am. Med. Assoc. 2021, 325, 669. 10.1001/jama.2021.0106 33591350

[5] S. J. Dixon , K. M. Lemberg , M. R. Lamprecht , R. Skouta , E. M. Zaitsev , C. E. Gleason , D. N. Patel , A. J. Bauer , A. M. Cantley , W. S. Yang , B. Morrison , B. R. Stockwell , Cell 2012, 149, 1060. 22632970 10.1016/j.cell.2012.03.042 PMC3367386

[6] a) L. Jiang , N. Kon , T. Y. Li , S. J. Wang , T. Su , H. Hibshoosh , R. Baer , W. Gu , Nature 2015, 520, 57; 25799988 10.1038/nature14344 PMC4455927

[7] T. J. Zheng , Q. B. Liu , F. Y. Xing , C. Zeng , W. D. Wang , J. Exp. Clin. Cancer Res. 2023, 42, 4. 37259067 10.1186/s13046-023-02712-2 PMC10230712

[8] a) J. Conde , N. Oliva , Y. Zhang , N. Artzi , Nat. Mater. 2016, 15, 1128; 27454043 10.1038/nmat4707 PMC6594055

[9] M. Drefs , M. B. Schoenberg , T. S. Schiergens , M. Albertsmeier , J. Andrassy , M. K. Angele , C. B. Westphalen , M. Seidensticker , J. Werner , M. O. Guba , J. Surg. Oncol. 2021, 123, 1578. 33684241 10.1002/jso.26443

[10] H. Huang , X. R. Wang , W. L. Wang , X. Y. Qu , X. J. Song , Y. W. Zhang , L. P. Zhong , D. P. Yang , X. C. Dong , Y. X. Zhao , Biomaterials 2022, 280, 11. 10.1016/j.biomaterials.2021.121289 34861512

[11] C. J. Brown , M. J. Raval , Expert Rev. Anticancer Ther. 2008, 8, 111. 18095888 10.1586/14737140.8.1.111

[12] E. D. Miller , K. E. Hitchcock , P. B. Romesser , J. Gastrointest. Cancer 2023, 54, 1116. 36652155 10.1007/s12029-022-00900-5 PMC10352468

[13] X. Sun , X. Liang , Y. Wang , P. Ma , W. Xiong , S. Qian , Y. Cui , H. Zhang , X. Chen , F. Tian , Y. Shi , F. Zheng , L. Li , Biomaterials 2023, 301, 122263. 37549506 10.1016/j.biomaterials.2023.122263

[14] N. W. Nkune , C. A. Kruger , H. Abrahamse , Anti‐Cancer Agents Med. Chem. 2021, 21, 137. 10.2174/1871520620666200415102321 32294046

[15] Y. Xu , Y. Liu , X. Hu , R. Qin , H. Su , J. Li , P. Yang , Angew. Chem., Int. Ed. Engl. 2020, 59, 2850. 31802603 10.1002/anie.201912848

[16] R. R. Qin , Y. S. Guo , H. Ren , Y. C. Liu , H. Su , X. Y. Chu , Y. Y. Jin , F. Lu , B. L. Wang , P. Yang , ACS Cent. Sci. 2022, 8, 705. 35756378 10.1021/acscentsci.2c00151 PMC9228557

[17] X. T. Fan , J. Lim , Z. B. Li , T. T. Wang , L. Jiang , S. Q. Liu , L. L. Zhou , C. B. He , J. Mater. Chem. B 2021, 9, 3509. 33909733 10.1039/d1tb00191d

[18] G. Y. Jiang , Z. B. Hu , L. Bai , C. Zhong , S. Lu , B. S. Han , Z. R. Sun , S. J. Zhu , Y. Y. Liang , H. T. Sun , J. Mater. Chem. C 2023, 11, 7243.

[19] N. N. Zheng , Y. T. Chen , L. Jiang , H. C. Ma , Colloids Surf., A 2022, 634, 8.

[20] C. Wu , Z. X. Wang , H. X. Lei , W. Zhang , Y. Duan , J. Am. Chem. Soc. 2007, 129, 1225. 17263405 10.1021/ja0662772

[21] Y. C. Liu , S. T. Miao , H. Ren , L. H. Tian , J. Zhao , P. Yang , Nat. Protoc. 2023, 37. 10.1038/s41596-023-00918-4 38049624

[22] W. Zhang , M. J. Czupryn , Biotechnol. Progr. 2002, 18, 509. 10.1021/bp025511z 12052067

[23] B. B. Koleva , Bulg. Chem. Commun. 2008, 40, 456.

[24] D. H. Wang , Y. Ha , J. Gu , Q. Li , L. L. Zhang , P. Yang , Adv. Mater. 2016, 28, 7414. 27337177 10.1002/adma.201506476

[25] J. L. Li , J. H. Tian , Y. T. Gao , R. R. Qin , H. M. Pi , M. J. Li , P. Yang , Chem. Eng. J. 2021, 410, 12.

[26] B. Z. Dong , X. H. Guo , K. D. Zhang , Y. P. Zhang , Z. H. Li , W. S. Wang , C. Cai , Surf. Coat. Technol. 2022, 429, 12.

[27] L. Patnaik , S. R. Maity , S. Kumar , Ceram. Int. 2020, 46, 22805.

[28] Y. W. Li , F. B. Kong , Compr. Rev. Food Sci. Food Saf. 2022, 21, 3804. 35880687 10.1111/1541-4337.13007

[29] Y. Z. Xu , S. Y. Liu , L. L. Zeng , H. S. Ma , Y. F. Zhang , H. H. Yang , Y. C. Liu , S. Fang , J. Zhao , Y. S. Xu , C. R. Ashby , Y. L. He , Z. Dai , Y. H. Pan , Adv. Mater. 2022, 34, 13.

[30] S. K. Ng , J. P. M. Wood , G. Chidlow , G. G. Han , T. Kittipassorn , D. J. Peet , R. J. Casson , Clin. Exp. Ophthalmol. 2015, 43, 367. 25330055 10.1111/ceo.12462

[31] C. X. Cui , Y. M. Fang , B. Q. Chen , T. W. Tan , Catal. Sci. Technol. 2019, 9, 477.

[32] K. J. Liu , H. L. Yao , Y. Wen , H. Zhao , N. J. Zhou , S. L. Lei , L. Xiong , Biochim. Biophys. Acta, Mol. Basis Dis. 2018, 1864, 2871. 29807108 10.1016/j.bbadis.2018.05.020

[33] X. R. Duan , H. L. Tian , S. W. Zheng , J. M. Zhu , C. Li , B. He , L. Li , H. Jiang , S. J. Lu , Y. M. Feng , G. T. Bentley , W. Zhang , C. H. Huang , W. Gao , N. Xie , K. Xie , Adv. Healthcare Mater. 2023, 12, 13.

[34] J. Pathak , A. Chatterjee , S. P. Singh , R. P. Sinha , Bio‐Protocol 2017, 7, e2545. 34541194 10.21769/BioProtoc.2545 PMC8413551

[35] a) L. Moretti , K. W. Kim , D. K. Jung , C. D. Willey , B. Lu , Mol. Cancer Ther. 2009, 8, 1270; 19417149 10.1158/1535-7163.MCT-08-0893 PMC2888880

[36] P. X. Wang , X. X. Cao , Y. Chu , P. X. Wang , Colloids Surf., B 2019, 181, 910. 10.1016/j.colsurfb.2019.06.050 31382340

[37] M. M. Chen , F. C. Yang , X. Chen , R. R. Qin , H. M. Pi , G. J. Zhou , P. Yang , Adv. Mater. 2021, 33, 11. 10.1002/adma.202104187 34510560

[38] L. Yang , J. N. Gao , Y. Q. Zhang , E. A. Perez , Y. C. Wu , T. N. Guo , C. Li , H. Wang , Y. Xu , J. Gastrointest. Oncol. 2023, 14, 2436. 38196536 10.21037/jgo-23-860 PMC10772689

[39] V. A. Davis , R. N. Persidskaia , L. M. Baca‐Regen , N. Fiotti , B. G. Halloran , B. T. Baxter , J. Surg. Res. 2001, 101, 152. 11735270 10.1006/jsre.2001.6281

[40] R. M. Hoffman , Invest. New Drugs 1999, 17, 343. 10759402 10.1023/a:1006326203858

[41] P. D. Leiphrakpam , A. J. Lazenby , L. M. Smith , M. G. Brattain , C. Are , J. Surg. Oncol. 2021, 123, 1764. 33765336 10.1002/jso.26464

[42] B. P. Jing , R. J. Qian , D. W. Jiang , Y. K. Gai , Z. Liu , F. Guo , S. Ren , Y. Gao , X. L. Lan , R. An , J. Nanobiotechnol. 2021, 19, 13. 10.1186/s12951-021-00888-3 PMC8141172 34022897

[43] S. Gerstberger , Q. W. Jiang , K. Ganesh , Cell 2023, 186, 1564. 37059065 10.1016/j.cell.2023.03.003 PMC10511214

[44] X. Zhang , T. Wu , X. Y. Cai , J. H. Dong , C. F. Xia , Y. C. Zhou , R. Ding , R. F. Yang , J. Tan , L. J. Zhang , Y. Zhang , Y. Q. Wang , C. Dong , Y. F. Li , Front. Immunol. 2022, 13, 13. 10.3389/fimmu.2022.795972 PMC8968082 35371084

[45] H. Ren , H. Chen , Y. Kang , W. Liu , Y. C. Liu , F. Tao , S. T. Miao , Y. Y. Zhang , Q. Liu , M. D. Dong , Y. G. Liu , B. Liu , P. Yang , Chem. Sci. 2024, 15, 8946. 38873054 10.1039/d4sc00560k PMC11168098

