# Supplementary material for: Functionalized Amyloid‐Like Protein Nanofilm‐Mediated Synergistic Disulfidptosis and Photodynamic Therapy for Preventing Postoperative Recurrence of Colorectal Cancer
Source: Small Sci. 2024 Sep 23;4(11):2400323. doi: 10.1002/smsc.202400323 (PMC11934982; doi:10.1002/smsc.202400323)
Supplement: Supplementary file 1 — Supplementary Material [file SMSC-4-2400323-s001.pdf]

Functionalized Amyloid-like Protein Nanofilm Mediated Synergistic Disulfidptosis and Photodynamic Therapy for Preventing Postoperative Recurrence of Colorectal Cancer

Man Zhang[#], Ke Li[#], Junhao Kou, Guozhi Lu, Ling Qiu, Chunzhao Yang, Yongchun Liu, Qi Xue*, Peng Yang**

M . Zhang, L . Qiu, C . Yang, Q . Xue
Southern Medical University Hospital of Integrated Traditional Chinese and Western Medicine, Southern Medical University, Department of General Surgery, Guangdong, 510315, China
K . Li
Xi'an Key Laboratory for Prevention and Treatment of Common Aging Diseases. Translational and Research Centre for Prevention and Therapy of Chronic Disease. Institute of Basic and Translational Medicine, Xi'an Medical University, Xi'an, 710021, China
J . Kou
College of Pharmacy, Xi'an Medical University, Xi'an, 710021, China
G . Lu
College of Clinical Medicine, Xi'an Medical University, Xi'an, 710021, China
Y . Liu, P . Yang
Key Laboratory of Applied Surface and Colloid Chemistry, Ministry of Education, School of Chemistry and Chemical Engineering, Shaanxi Normal University, Xi'an, 710119, China
E-mail: surfbioliu@snnu.edu.cn (Y. Liu)
E-mail: xueqi_doctor@126.com (Q . Xue)
E-mail: yangpeng@snnu.edu.cn (P . Yang)

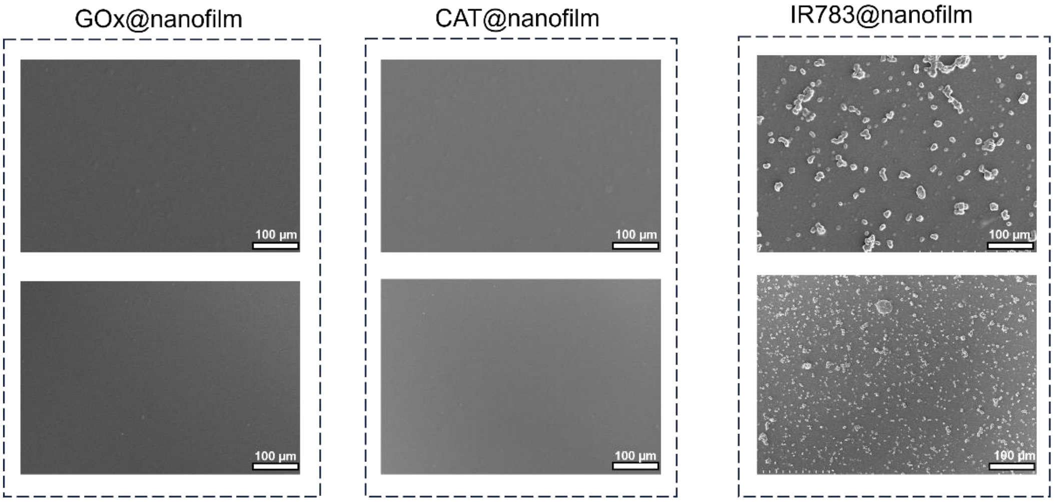

Figure S1. SEM images of GOx@nanofilm, CAT@nanofilm, and IR783@nanofilm formed by separately adding GOx, CAT, and IR783 into the lysozyme-cysteine film formation system, scale=100 μm

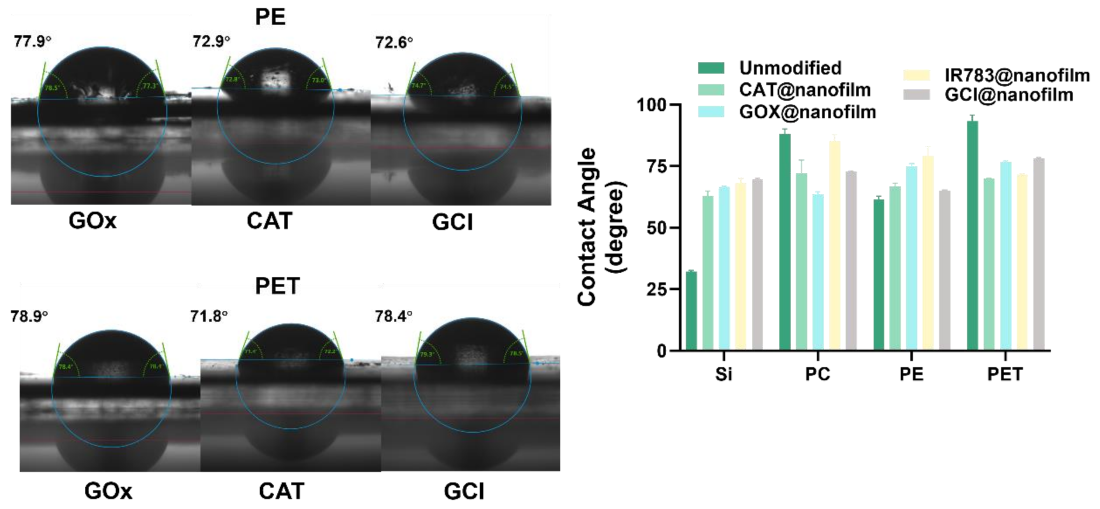

Figure S2. Water contact angles of unmodified substrates and substrates modified with GOx@nanofilm, CAT@nanofilm, IR783@nanofilm, and GCI@nanofilm. Si, silicon wafer; PC, polycarbonate; PE, polyethylene; PET, polyethylene terephthalate.

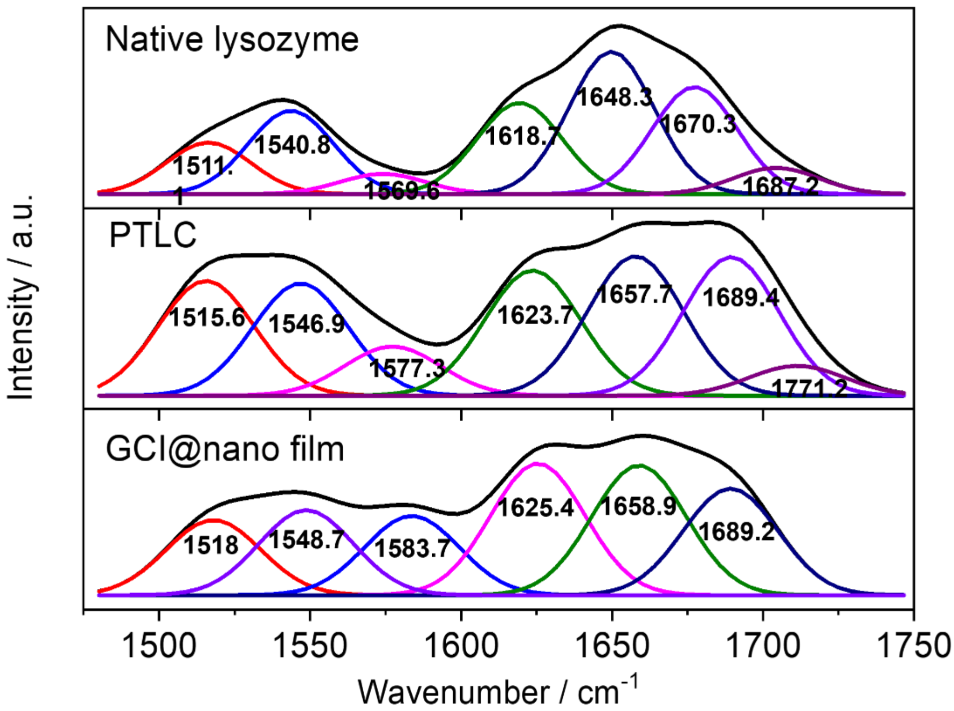

Figure S3. IR spectra deconvolution of amide I and amide II bonds for native lysozyme, PTLC, and GCI@nanofilm, showing changes in β-sheet content.

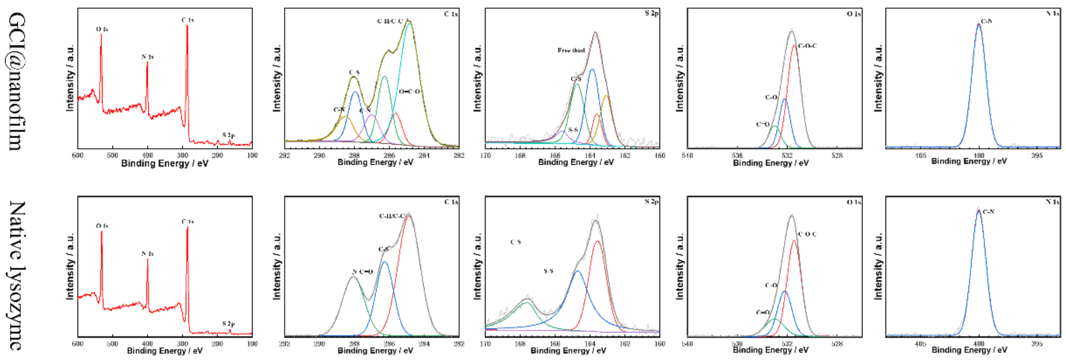

Figure S4. XPS wide-scan spectra of GCI@nanofilm and native lysozyme, along with their corresponding high-resolution spectra of C1s, S2p, O1s, and N1s.

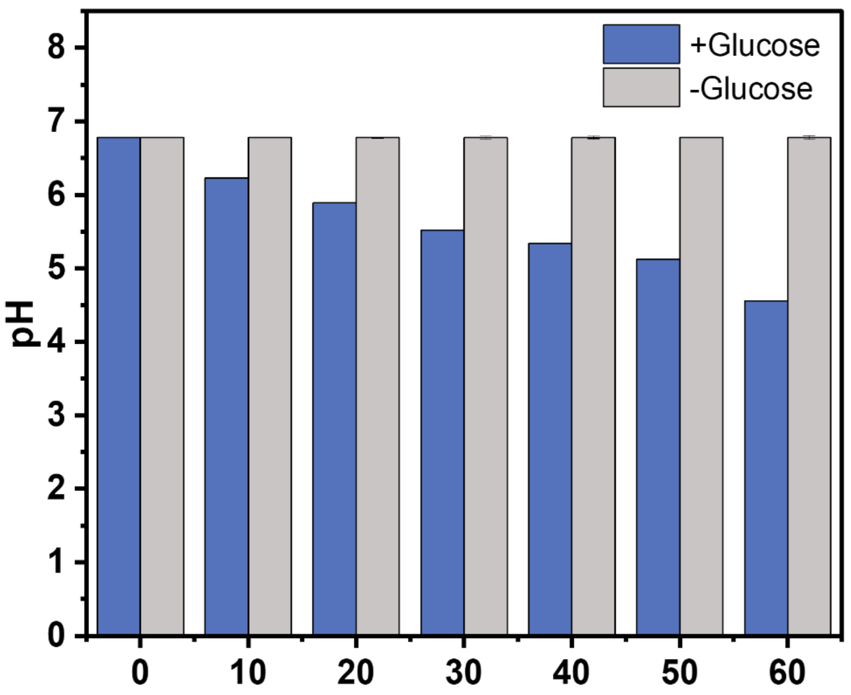

Figure S5. Effect of GCI@nanofilm on pH with or without 5 μM glucose solution.

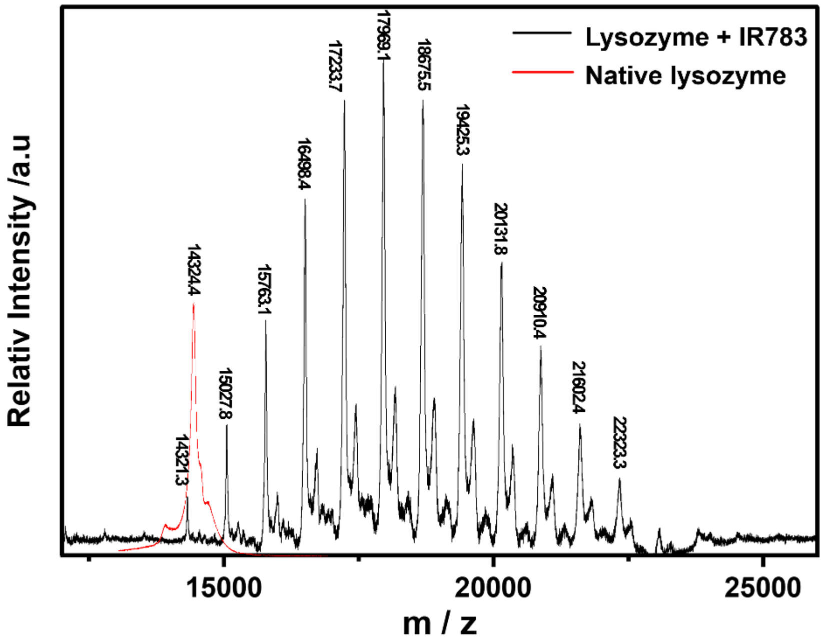

Figure S6. Mass spectrum of lysozyme grafted with IR783

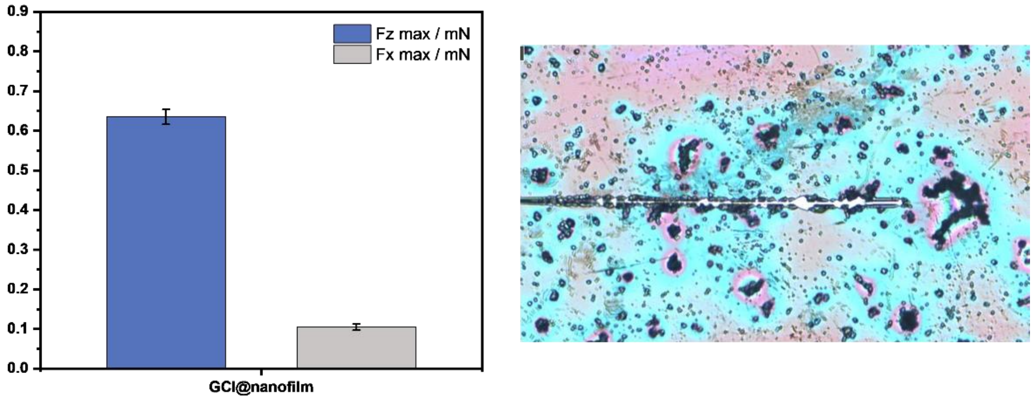

Figure S7. Normal force (Fz)and tangential force (Fx) determined for the GCI@nanofilm during the micro-scratch rising force test. Nano-scratch instrument probe r=2 µm.

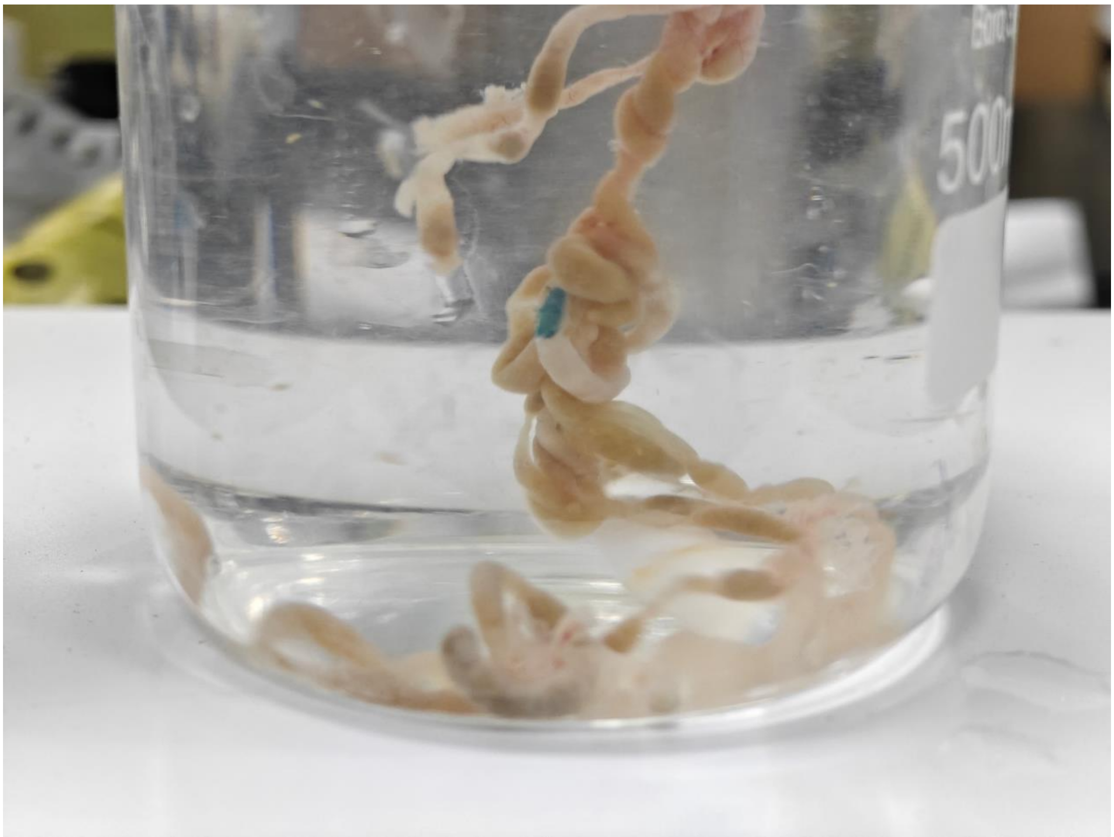

Figure S8. Ex vivo intestinal experiments with GCI@nanofilm-adhered mice.

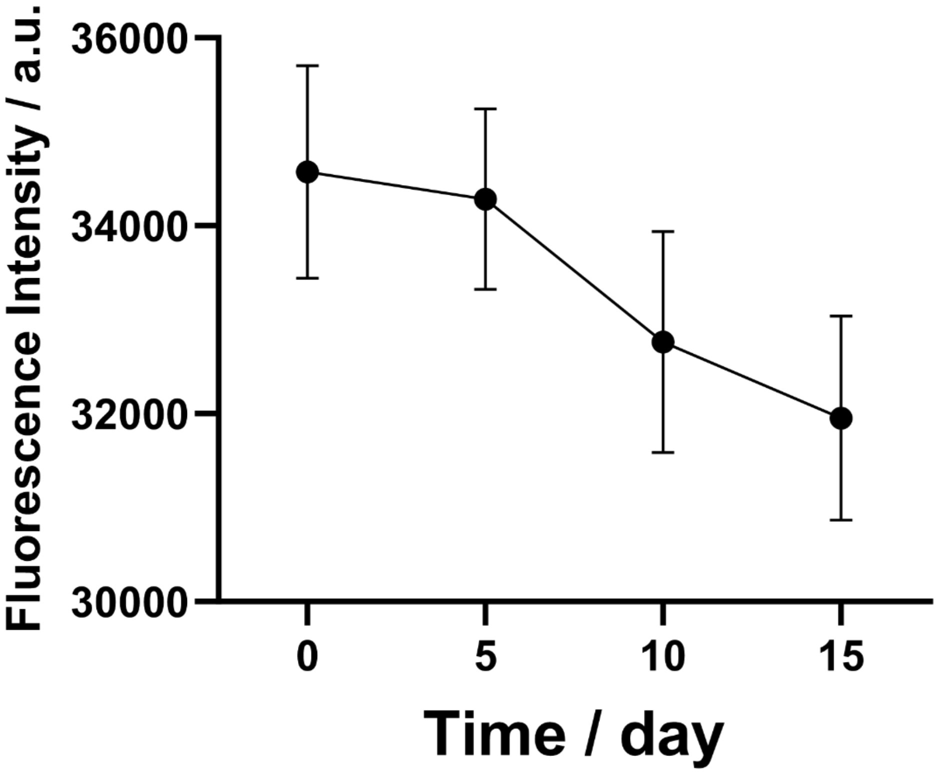

Figure S9. Fluorescence intensity of GCI@nanofilm adhered to the intestine over time, indicating the adhesion stability of GCI@nanofilm

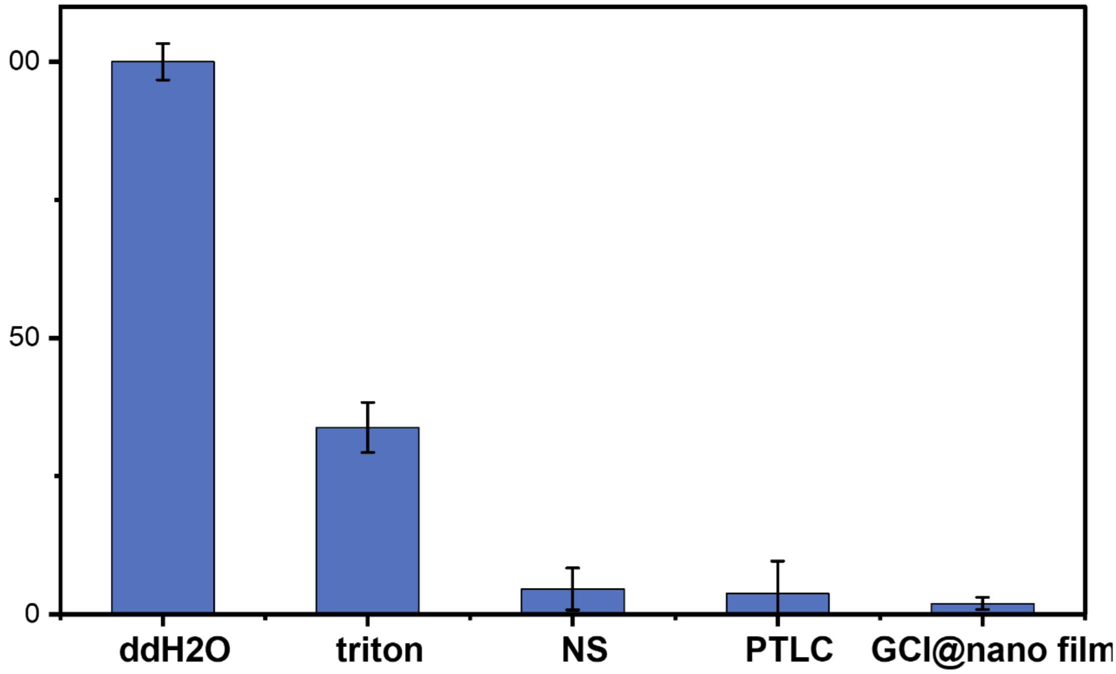

Figure S10. Hemolysis assay of PTLC and GCI@nanofilm.

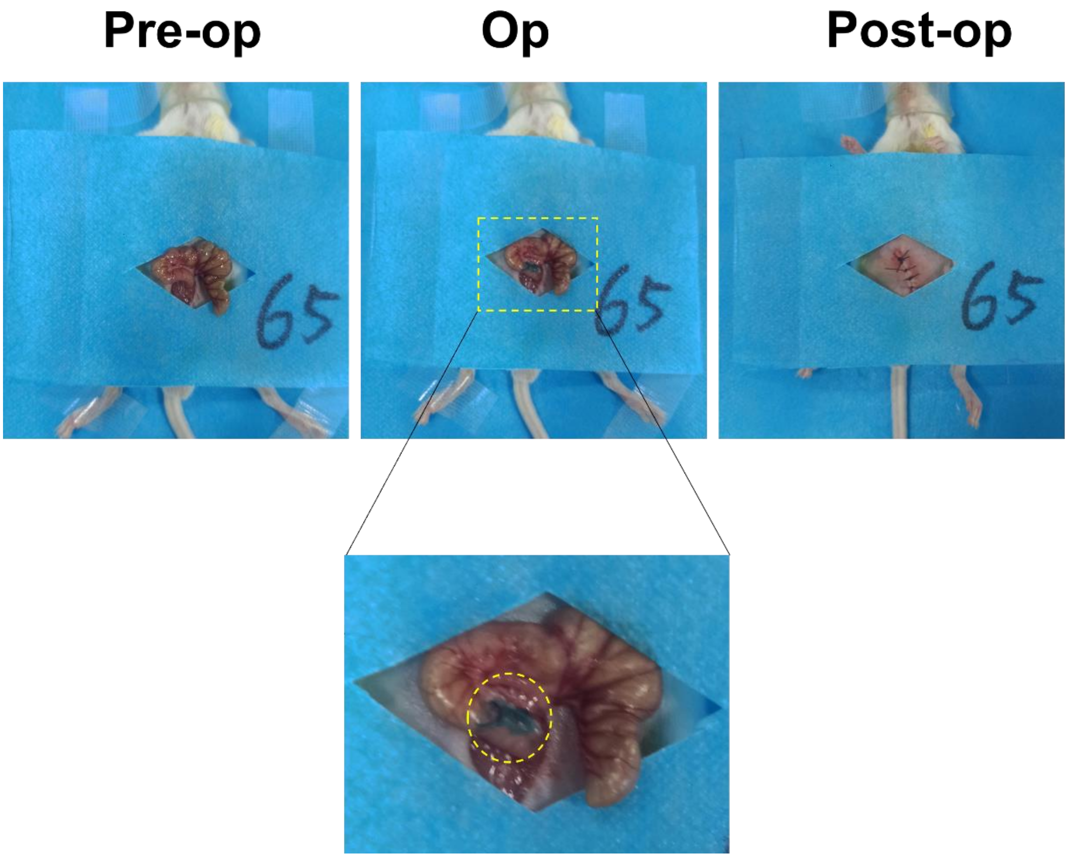

Figure S11. Procedures and optical images of mice with GCI@nanofilm adhesion.

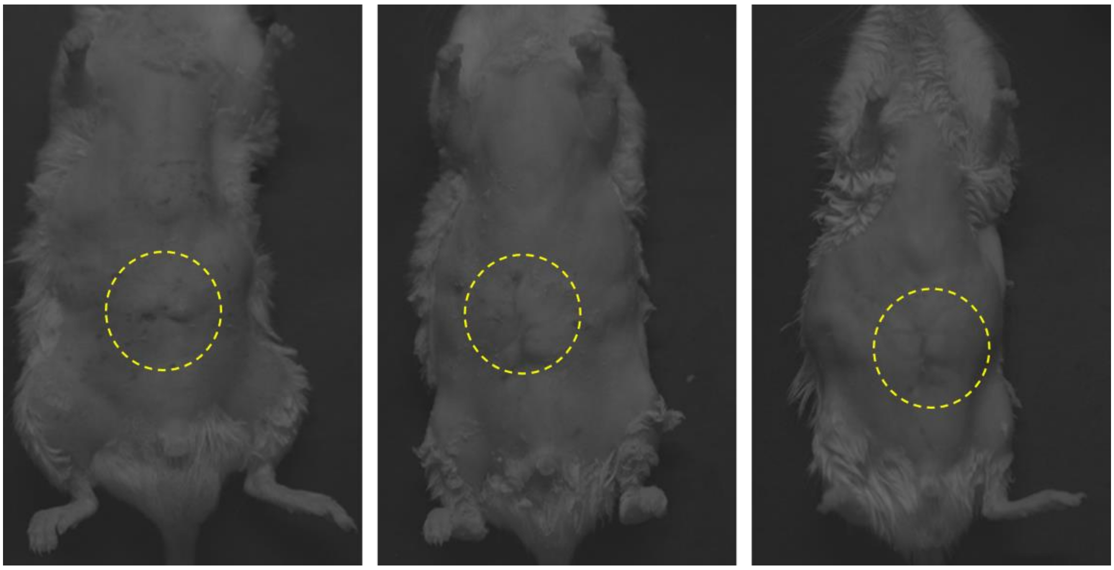

Figure S12. In vivo fluorescence imaging of mice with GCI@nanofilm adhesion after 6 weeks, showing degradation of GCI@nanofilm.

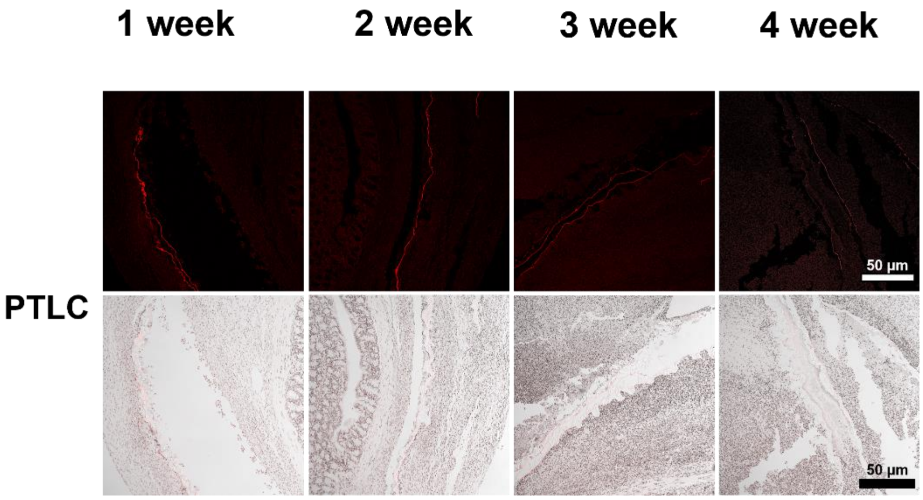

Figure S13. Confocal images showing in situ degradation of IR783-grafted PTLC film in the colorectal region, scale = 50 μm.

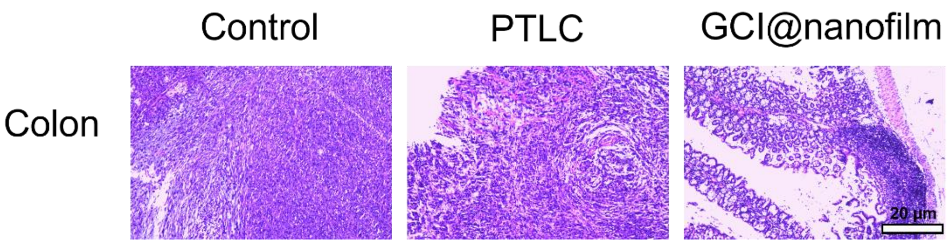

Figure S14. HE staining images of in situ colon tumors in mice from the control, PTLC, and GCI@nanofilm groups after 14 days of treatment, scale = 20 μm.

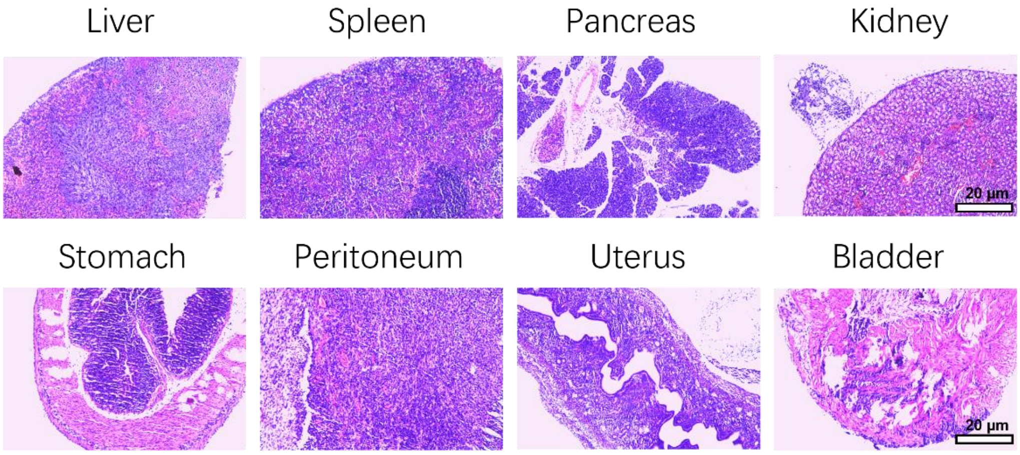

Figure S15. Pathological sections of major organs in the control group after 14 days, showing liver metastasis and suspected malignancy in the kidney capsule, with no cancer tissue found in other organs, scale = 20 μm.
